# Real‐Time Neurofeedback Training to Modulate Glutamate‐Glutamine Concentration Using Functional Magnetic Resonance Spectroscopy

**DOI:** 10.1002/nbm.70399

**Published:** 2026-09-21

**Authors:** Zhiyan Wang, Savanna Babu, Nina Beck, Sebastian M. Frank

**Affiliations:** ^1^ Institute for Experimental Psychology University of Regensburg Regensburg Germany

**Keywords:** excitatory processing, functional magnetic resonance spectroscopy, glutamate, glutamine, Glx, human participants, neurofeedback, occipital lobes, point resolved spectroscopy, training

## Abstract

Neurofeedback training is a procedure in which participants learn to modulate brain activity through their own efforts. Here, we designed a real‐time neurofeedback training using functional magnetic resonance spectroscopy (fMRS) to modulate the concentration of glutamate, a major excitatory neurotransmitter, and its precursor glutamine (collectively referred to as Glx) in a volume‐of‐interest (VOI) located in the occipital lobes. During an induction phase participants employed a self‐selected strategy to increase or decrease Glx concentration relative to a reference concentration measured prior to neurofeedback training. Participants were informed about their performance after the induction phase by displaying a disk in green for success or red for failure. The disk size indicated the magnitude of success or failure. The procedure was repeated over the course of 30 trials. Participants were randomly assigned to experimental and control groups (15 participants per group). The experimental group received veridical feedback about their success or failure in the induction phase. The control group received random feedback. Glx concentration in the VOI was measured using 2‐s‐long consecutive Point RESolved Spectroscopy (PRESS) scans in a 3‐Tesla MRI scanner. The results showed that depending on group assignment, participants in the experimental group learned to increase or decrease Glx concentration over the course of training. No significant changes in Glx concentration were observed in the control group. These findings suggest that, with the help of veridical performance feedback, participants learned to modulate Glx concentration in the VOI in the desired direction over a relatively short period of time. fMRS‐based neurofeedback training may complement existing neurofeedback approaches by targeting neurotransmitter‐related processes.

## Graphical abstract

Neurofeedback training with functional magnetic resonance spectroscopy to modulate glutamate‐glutamine (Glx) concentration in the occipital lobes. Participants learned to induce an increase or decrease in Glx concentration with a self‐selected strategy using real‐time feedback about their success or failure.

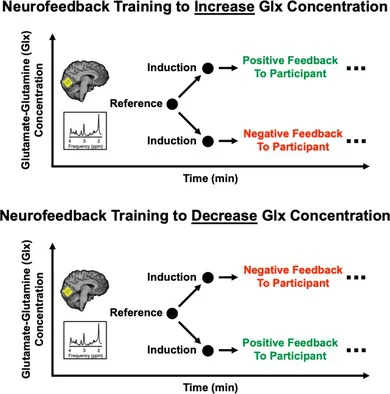

## 1 Introduction

Neurofeedback training is used to non‐invasively modulate brain activity in real time, relying on participants' own efforts [1, 2, 3, 4]. The goal is to achieve certain patterns of activity in the whole brain or in selected regions of interest [5, 6, 7, 8, 9, 10, 11, 12, 13]. Procedures for neurofeedback training have been developed for the treatment of patients with attention deficit disorders [14, 15], anxiety disorders [16, 17, 18], sleep problems [19, 20], or chronic pain [21, 22, 23]. Even if the effectiveness of neurofeedback training is not without controversy [24, 25, 26], it is still considered a useful tool to provide additional support to the treatment of patients [27] and to augment neural processing in healthy participants [2, 4, 28].

A typical procedure for neurofeedback training includes the following steps (see [2, 4, 10, 28, 29, 30, 31]): First, activity is measured before neurofeedback training as a reference. Then, in each training trial, participants attempt to modulate activity during an induction phase using a self‐selected strategy. The neurofeedback training paradigm is a mind‐wandering task in which the only difference between reference and induction phases lies in the content of participants' thoughts, that is, in the strategy they use to modulate activity during the induction phase. The achieved activity in the induction phase is transferred to an analysis computer in real‐time and compared with the reference activity. Participants see a visualization (e.g., a disk with variable size, a video game, or an animation) on a screen that changes depending on the achieved activity change. The goal is to modulate (e.g., increase) activity relative to the reference. The feedback (i.e., size of the disk, progress in the game, or change in animation) shows participants how well they are achieving the goal.

Common ways to measure brain activity for neurofeedback training are electroencephalography (EEG) or functional magnetic resonance imaging (fMRI) [2, 4, 9, 14]. However, the potential of functional magnetic resonance spectroscopy (fMRS) for neurofeedback training has not yet been explored. fMRS is an MRI‐based imaging technique that measures changes in metabolite concentrations, including glutamate, a major excitatory neurotransmitter, and its precursor glutamine (collectively referred to as Glx), within a volume‐of‐interest (VOI) across experimental conditions [32, 33, 34, 35, 36, 37]. fMRS may be useful as a complementary method to EEG and fMRI, as it has the potential to provide information about neurochemical processes rather than only electrophysiological or hemodynamic activity. Relative to EEG, fMRS offers greater anatomical specificity (albeit with limitations due to the large VOI size that is typically in the range of cm in fMRS) and access to metabolite‐related changes that are not directly observable in scalp‐recorded signals. Relative to fMRI, it may add a more mechanistic perspective by capturing changes in neurotransmitter‐related processes. Altered glutamate‐glutamine concentrations, as assessed by MRS, have been reported in various clinical conditions, such as dementia [38, 39], depression [40], epilepsy [41], and schizophrenia [42, 43], suggesting dysfunction in excitatory neurotransmitter‐related processes. An fMRS‐based neurofeedback training aimed at modulating Glx concentration in a desired direction through participants' own efforts may hold potential for addressing such alterations.

A previous study showed that fMRS is in principle feasible for neurofeedback [44], but a procedure for real‐time neurofeedback training to modulate Glx concentration has not yet been established. Here, we developed a procedure for real‐time neurofeedback training with fMRS to increase or decrease Glx concentration in a VOI placed in early visual areas (V1 and V2) in the occipital lobes. Two experiments were conducted in healthy young adults. In the first experiment, participants trained to increase Glx concentration through their own efforts. They were randomly assigned to an experimental group with veridical feedback about their success or failure in each induction trial of neurofeedback training and a control group with random feedback. The control group was included to account for variations in Glx concentration associated with task performance, as well as for random fluctuations in Glx levels over time. In the second experiment, participants trained to decrease Glx concentration through their own efforts. We hypothesized that participants would learn to modulate Glx concentration in the desired direction over the course of neurofeedback training, whereas no changes in Glx concentration were expected in the control group.

## 2 Methods

### 2.1 Participants

Thirty‐six healthy participants (22 females, 14 males, mean ± standard‐error‐of‐the‐mean [SEM] age = 23.6 ± 0.66 years old; 34 right‐handed, 2 left‐handed) with normal or corrected‐to‐normal vision were recruited. In the first experiment, 30 participants were randomly assigned to one of two groups (the experimental group and the control group with 15 participants each). The number of participants per group followed previous fMRI‐based neurofeedback training studies focusing on early visual areas [10, 12, 45]. A subset of nine participants from the first experiment volunteered for a second follow‐up experiment. In order to keep the number of participants equal to the experimental group in the first experiment, six new participants were additionally recruited for the second experiment. Participants gave informed written consent and received course credit or monetary compensation for their participation. Participants (except two experimenters who also participated) were naïve to the experimental design. The study was approved by the internal review board of the University of Regensburg.

### 2.2 Neurofeedback Training

**FIGURE 1:**
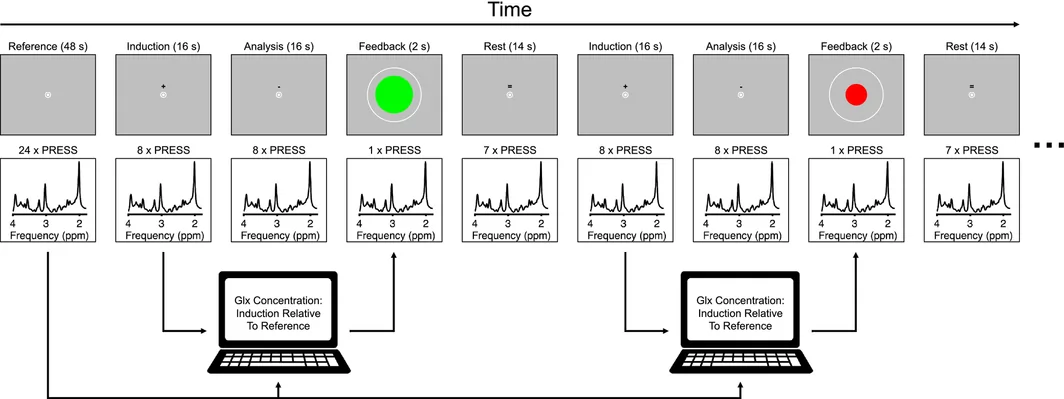
Neurofeedback training with functional magnetic resonance spectroscopy (fMRS). The procedure commenced with the collection of a reference scan to measure Glx (glutamate + glutamine) concentration prior to neurofeedback training. Then, during an induction phase (plus sign above central fixation), participants attempted to increase or decrease Glx concentration using a self‐selected strategy, followed by an analysis phase (minus sign above central fixation). During a feedback phase, participants were informed about the achieved change in Glx concentration relative to the reference concentration by changing the color and size of a central disk. An achieved change in the desired direction was visualized as a green disk. A failure was visualized as a red disk. The larger the disk, the greater the achieved change. Feedback was displayed for 2 s, followed by a rest period with an equals sign above central fixation for 14 s before the next neurofeedback training trial commenced.

A neurofeedback training was developed for single‐voxel proton (^1^H) fMRS conducted with a Prisma 3 Tesla MRI scanner (Siemens, Erlangen, Germany; MRI scanner software: Syngo MR XA30) using a head/neck coil with 64 channels. The core principles of this procedure are based on similar neurofeedback trainings for fMRI (e.g., [12, 45, 46]). Figure 1 shows an overview of the procedure with fMRS. A checklist containing all fMRS acquisition parameters, analyses, and quality checks [47], is provided in Table S1. fMRS measurements were conducted with Point RESolved Spectroscopy (PRESS; [48, 49]) scans [time‐to‐repeat (TR) = 2 s; time‐to‐echo (TE) = 30 ms; flip angle (FA) = 90°] using WET water suppression [50].

**FIGURE 2:**
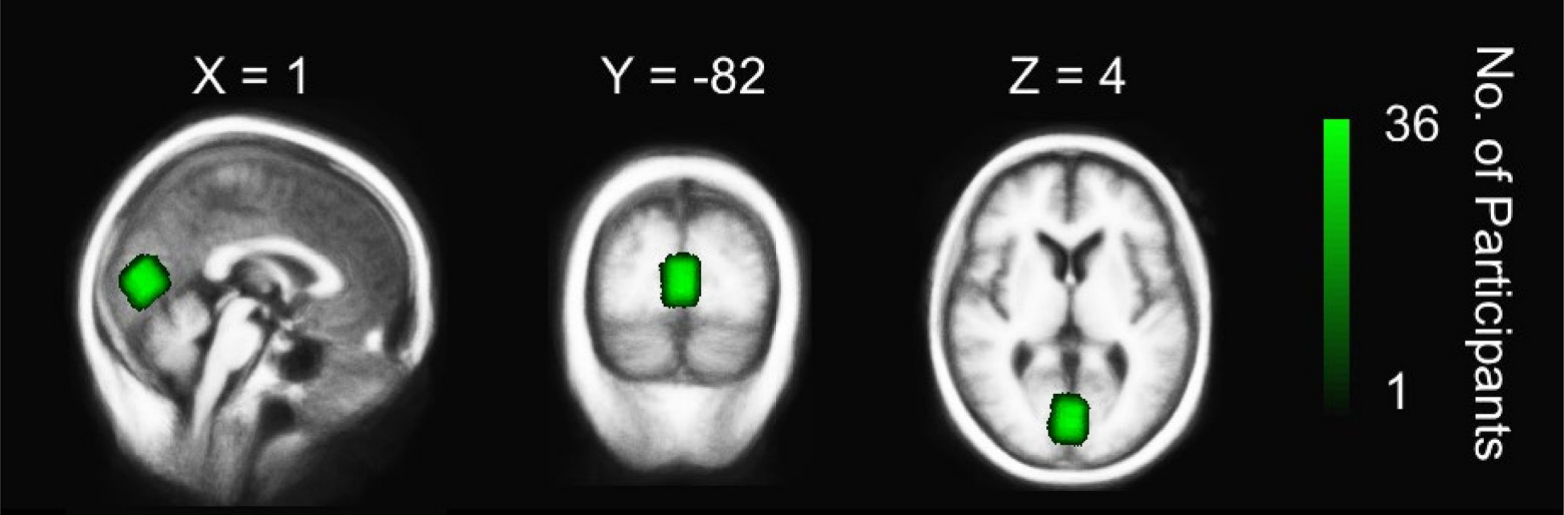
Volume‐of‐interest (VOI) location across participants. Brighter colors correspond to greater overlap across participants. The VOI location is overlaid on a standard brain. *X*, *Y*, and *Z* correspond to mean MNI coordinates of the center of the VOI across participants. No. = number.

Participants completed a total of 30 neurofeedback training trials, divided into two consecutive fMRS runs. The first fMRS run consisted of the following five steps. First, for the purpose of VOI placement, three short high‐resolution anatomical scans for the sagittal, coronal, and transverse planes were collected. The scans had the following imaging parameters: sagittal plane: TR = 0.19 s, TE = 2.46 ms, FA = 70°, in‐plane acquisition matrix (AM) = 288 × 288, 25 slices, voxel size = 0.8 × 0.8 × 4.0 mm, inter‐slice gap = 1.20 mm; coronal plane: TR = 0.25 s, TE = 2.46 ms, FA = 70°, AM = 288 × 288, 35 slices, voxel size = 0.8 × 0.8 × 4.0 mm, inter‐slice gap = 1.20 mm; transverse plane: TR = 0.19 s, TE = 2.46 ms, FA = 70°, AM = 288 × 288, 27 slices, voxel size = 0.8 × 0.8 × 4.0 mm, inter‐slice gap = 1.20 mm. Second, using the anatomical scans, the VOI (size: 2 × 2 × 2 cm) was placed perpendicular to the calcarine sulcus and centered between the occipital lobes, similarly to Frank et al. [51]. Non‐neural tissue containing lipids and proximity to the dura were avoided during VOI placement. The VOI had the following mean ± SEM MNI coordinates across participants: *X* = 1.04 ± 0.35; *Y* = −81.8 ± 0.46; *Z* = 3.95 ± 0.73 (Figure 2). Automated shimming was performed. Scanning continued only if the shim value corresponding to the linewidth of the unsuppressed water peak was below 20 Hz (which was the case for every participant). Third, a PRESS scan with 16 averages without water suppression (water reference scan) was collected (scan time: 42 s, including four preparation scans). Fourth, the Glx reference concentration (i.e., the concentration prior to neurofeedback training) was measured with a PRESS scan with 24 averages (scan time: 48 s). During the collection of this scan a bull's eye was displayed at central fixation (diameter: 0.8°). Participants were instructed to maintain fixation on the bull's eye throughout the experiment. They received no further instructions for this initial phase of the experiment, except that they could relax, meaning that they were free to let their minds wander in any direction as they wished, and were not informed of its purpose. Fifth, 15 neurofeedback training trials were conducted consecutively. Each trial consisted of four consecutive phases (Figure 1).

In the first phase of a neurofeedback training trial (“Induction”) a plus sign was displayed above the bull's eye. During this phase, participants attempted to make the subsequently presented disk green and large (i.e., unknown to them, increase or decrease Glx concentration relative to the reference concentration in the VOI). During the induction phase a PRESS scan with eight averages was collected (scan time: 16 s). In the second phase of the trial (“Analysis”), a minus sign was displayed above the bull's eye. Participants were instructed that as soon as they saw the minus symbol, they should stop their efforts and rest (i.e., they were free to let their minds wander in any direction as they wished). In this phase, the spectrum collected in the induction phase was analyzed and the feedback on participants' success or failure was calculated (see Section 2.6 below). A PRESS scan with eight averages was collected during the analysis phase (scan time: 16 s). This time window was necessary in our technical setup to carry out the analysis steps and was comparable to durations used in fMRI‐based neurofeedback trainings (e.g., [52]). In the third phase of the trial (“Feedback”), participants received feedback about their success or failure in the induction phase. For this purpose, a disk was displayed within a circle (circle diameter: 10°). The color of the disk indicated whether the induction was successful or not. If it was successful (i.e., participants achieved a change in Glx concentration in the desired direction during the induction), the disk color was green. Otherwise it was red. The size of the disk indicated the magnitude of success or failure relative to a predefined goal, which was adjusted based on participants' performance over the course of neurofeedback training (see below). The larger the disk, the greater was the success or failure. For example, if participants achieved 100% or more of the goal, a green disk would completely fill the area within the circle. If they achieved 50% of the goal, a green disk would fill half the area within the circle. If they achieved −50% of the goal, a red disk would fill half the area within the circle, etc. If they achieved no change, no disk would be displayed within the circle. A two‐down‐one‐up staircase rule was used to adjust the goal based on participants' performance over the course of neurofeedback training. To this aim, three categories of induced Glx change were defined: first, large change in the desired direction (great success); second, small change in the desired direction (small success); and third, change in the opposite direction (failure). Great and small success were further defined as follows: For each trial, the desired magnitude of Glx change relative to the reference concentration was predefined as the goal participants should achieve. The initial goal at the beginning of the fMRS run was defined as 20% change in Glx concentration in the desired direction relative to the reference concentration. How well participants achieved this goal was reflected in the disk size during the feedback phase. If participants achieved 50% or more of the goal, the induction trial was classified as a large change (great success). If participants achieved 0%–50% of the goal, the induction trial was classified as a small change (small success). If participants achieved a great success in two consecutive trials, the goal for the next trial was increased with a log unit of 0.2 relative to the previous goal or the initial goal (20%). As a result, the change of disk size in the next trial with great/small success became smaller, encouraging participants to further optimize their strategies. If participants had a small success in a trial, the goal was decreased with a log unit of 0.2 relative to the previous goal or initial goal (20%) and the change of disk size in the next trial with great/small success became larger. If participants failed to induce a change in the desired direction in a trial, no adjustment was made to the change of disk size in the next trial with great/small success or failure. Participants were instructed to use the feedback to optimize their strategies for subsequent trials. Feedback was displayed for 2 s. Finally, in the fourth phase of the trial (“Rest”) an equals sign was displayed above the bull's eye for 14 s, during which participants were instructed to relax (i.e., they were free to let their minds wander in any direction as they wished; note that participants may also have considered how to optimize their strategies for the next trial during this phase). A PRESS scan with eight averages was collected during feedback and rest phases (scan time: 16 s).

The procedure in the second fMRS run was identical to the first fMRS run, except that the first step (collection of short anatomical scans for VOI placement) was omitted because the same VOI location as in the first fMRS run was used. For one participant in the second experiment, only one fMRS run could be conducted due to time constraints. Due to a technical error, the results of one of the 30 induction trials were not recorded in three participants in the experimental group and one participant in the control group in the first experiment.

### 2.3 Participant Instruction for Neurofeedback Training

Participants received the following instructions by an experimenter blind to participants' assignments to experimental and control groups and experiments: “When a plus sign is presented above the bull's eye, please try to regulate your brain to try to make the disk green and as large as possible. You can think of anything that you think might work. If the disk gets larger and remains green, that means you are going in the right direction. You can keep thinking about what you have just thought. If the disk turns red, that means you are thinking in the wrong direction. The larger the red disk is, the further your thoughts are getting away from the ideal strategy. You can try to change your strategy. Try to make the disk green and larger and larger, if possible. When only a bull's eye is presented, or a bull's eye with a minus or an equals sign above it, try to relax but remain fixated on the center of the bull's eye. You may let your mind wander freely in any direction. Please maintain central fixation throughout the entire experiment. Please try not to move your fingers or your head. Stick with the thoughts that go in the right direction and make the disk green. Try a different strategy if the disk turns red. You must find the best strategy for yourself. Everybody can have a different strategy.”

### 2.4 Experiments

Two experiments were conducted. In the first experiment, participants in the experimental group performed neurofeedback training to increase Glx concentration in the VOI. They received veridical feedback about their success or failure after each induction. Participants in the control group completed the same neurofeedback training but with random feedback (as described in [53]). To this end, the color and size of the disk in the feedback phase were random on each trial. The control group was included to account for variations in Glx concentration associated with performing the induction task irrespective of the veridicality of performance feedback, as well as for random fluctuations in Glx levels over time. The mean ± SEM number of trials with positive feedback across participants in the first experiment was 23.8 ± 0.96 for the experimental group and 15.5 ± 0.63 for the control group. In the second experiment, participants performed neurofeedback training to decrease Glx concentration in the VOI, receiving veridical feedback about their success or failure after each induction. The mean ± SEM number of trials with positive feedback across participants in the second experiment was 7.20 ± 1.25. Participants in each group and experiment received identical instructions for neurofeedback training. The subset of participants from the first experiment who also completed the second experiment was not informed about the change in neurofeedback training in the second experiment. After scanning, participants reported their strategies during induction.

### 2.5 Stimulus Presentation

Stimuli were generated using Psychtoolbox (Version 3.0.19; [54, 55]) running in MATLAB (Version 2022b; The Mathworks, Natick, MA, USA). Stimuli were displayed on a translucent screen located at the back of the scanner bore using a projector with gamma correction and a refresh rate of 60 Hz. Participants viewed the screen with a headcoil‐mounted mirror (viewing distance = 97 cm).

### 2.6 Real‐Time fMRS Analysis

A processing pipeline was developed with automatic steps for fMRS real‐time data export, analysis and feedback to participants. Immediately after an fMRS spectrum was collected, it was automatically exported from the Siemens scanner computer to a separate stimulation and analysis computer (see [4, 12, 45]). On this computer two instances of MATLAB ran in parallel. The first instance was used for visual stimulation. The second instance was used for fMRS analysis. Both instances were connected via the MATLAB tcp/ip interface from the instrument control toolbox. The data format of the spectrum exported from the scanner computer was Siemens DICOM. Prior to exporting, the spectrum was frequency‐ and phase‐corrected and averaged using Siemens' internal software. No additional frequency or phase correction was performed after export. On the stimulation and analysis computer the exported spectrum was converted to RAW format using Osprey (Version 2.4.0; [56]). The RAW file was submitted to fMRS analysis using the LC model (Version 6.3‐R; [57, 58]) running under the second instance of MATLAB. The spectrum was analyzed in the chemical shift range between 1.7 and 4.0 ppm. Water scaling and eddy current correction were performed using the water reference scan. The metabolite intensities were fitted to a linear combination of spectra of individual metabolites derived from an imported metabolite basis set [57, 58] (see Table S1). The concentration of Glx was normalized to the composite measure of N‐Acetylaspartate and N‐Acetylaspartylglutamate (NAA + NAAG) from the same spectrum by dividing the Glx concentration by the NAA + NAAG concentration [59, 60, 61]. The result (i.e., the normalized Glx concentration) was automatically transferred to the first instance of MATLAB. For induction scans, the percentage change of normalized Glx concentration relative to the normalized Glx concentration in the reference scan was calculated. The percentage change was positive if Glx concentration during induction was greater than the reference concentration. Otherwise, the percentage change was zero or negative. The direction and magnitude of the achieved change in Glx concentration was reported to participants by changing the color and adjusting the size of the disk displayed at the center of the screen during the feedback phase (see Figure 1 and *Neurofeedback Training* above).

### 2.7 fMRS Post Hoc Analyses

An increase in the blood‐oxygenation‐level‐dependent (BOLD) hemodynamic response may narrow the linewidth of the spectrum, thereby biasing estimates of neurotransmitter concentrations [33, 62, 63, 64, 65]. In order to account for BOLD‐induced linewidth differences between reference and induction spectra, we first quantified the linewidth difference (Δ full width at half maximum [FWHM]) using the NAA signal at 2.0 ppm. The mean ± SEM linewidth difference across induction trials and participants was 0.01 ± 0.03 Hz, suggesting that the BOLD effect was very small in our dataset and sometimes reversed, meaning that the reference spectrum showed narrower linewidth than the induction spectra. This is likely due to our study design in which participants were also free to let their minds wander during the reference period, which is different from a design comparing stimulation vs. baseline periods (e.g., visual checkerboard stimulation vs. blank as in the study by [62]). To correct for any linewidth differences between the reference and induction spectra, we performed Lorentzian linebroadening [33, 62, 63, 64, 65] to either the reference or induction spectra (depending on which had a narrower linewidth based on the ΔFWHM of NAA in the reference spectrum and mean induction spectrum across trials in each fMRS run) for each participant. The factor of Lorentzian linebroadening was set to a fixed value of 0.46 Hz, following the approach in Bednařík et al. [62]. The corrected spectra were analyzed using the LC model. See Figures [Link], [Link], [Link] for corrected spectra, and Figure S4 for difference spectra (induction minus reference) before and after correction using Lorentzian linebroadening.

fMRS data quality checks included (1) Cramer‐Rao Lower Bounds (CRLB) of estimated metabolite concentrations, (2) signal‐to‐noise (SN) ratio in the spectrum, and (3) FWHM of spectral linewidth (all derived from the LC model analysis). The mean ± SEM results across participants were as follows: CRLB across fMRS reference scans: 7.37% ± 0.07% (min: 6%; max: 10%) for Glx and 2.62% ± 0.06% (min: 2%; max: 4%) for NAA + NAAG. CRLB across fMRS induction scans: 7.98% ± 0.03% (min: 5%; max: 13%) for Glx and 3.21% ± 0.02% (min: 2%; max: 6%) for NAA + NAAG. SN ratio: 32.4 ± 0.70 (min: 17; max: 47) across fMRS reference scans and 21.7 ± 0.14 (min: 12; max: 35) across fMRS induction scans. FWHM: 5.16 ± 0.05 Hz (min: 4.07 Hz; max: 6.41 Hz) across fMRS reference scans and 5.12 ± 0.02 Hz (min: 3.57 Hz; max: 8.25 Hz) across fMRS induction scans.

Furthermore, for each participant, we examined whether any outlier values were present among the trial‐wise induced percent Glx concentration changes within a session, as such values may reflect motion artifacts. We used the three scaled median absolute deviation (MAD) away from the median as criterion to define outlier values. The scaling factor of the MAD is defined as $1/\Phi^{-1}\left(3/4\right)\approx 1.483$, in which $\Phi^{-1}$ is the inverse of the cumulative distribution function of the standard normal distribution. The MAD was calculated across all induction trials for each participant. In the first experiment, outlier induction trials were removed from three participants in the experimental group (one, two, and one trials, respectively) and four participants in the control group (one, one, one, and two trials, respectively). In the second experiment, outlier induction trials were removed from four participants (one, one, one, and three trials, respectively).

Each participant's high‐resolution anatomical scan of the brain was reconstructed using Freesurfer (Version 4.1.0; Martinos Center for Biomedical Imaging, Charlestown, MA, USA; [66, 67]). Freesurfer's segmentation of the brain into gray matter, white matter and other types of volume including cerebrospinal fluid was used for post hoc volume fraction analysis within the VOI. The mean ± SEM volume fractions in percent across participants were as follows: gray matter: 60.4% ± 0.97% (min: 50.3%; max: 71.8%); white matter: 33.1% ± 1.25% (min: 6.18%; max: 46.3%); other types of volume: 6.50% ± 1.08% (min: 0.64%; max: 38.5%).

To identify which visual areas were contained in the gray matter of the VOI, participants also completed an fMRI localizer scan (similar to the approach in [68]). In this scan, the vertical and horizontal meridians were stimulated with checkerboard patterns flickering in different colors (flicker frequency: 8 Hz). Two other visual field locations were also stimulated for the purpose of different studies. Each location was stimulated in a separate block lasting 12 s. For each location, three blocks were presented in random order. Each block with stimulation was followed by a 12‐s‐long baseline without stimulation. A central fixation spot was presented throughout. Participants completed a speeded dimming detection task at the fixation spot. In this task, the fixation spot briefly changed color in an unpredictable fashion. Participants pressed a button when they detected a color change. Their mean ± SEM response accuracy in the detection task was 98.6% ± 0.83% (min: 75%; max: 100%) correct. fMRI data were acquired with a T2*‐weighted echoplanar imaging (EPI) sequence (TR = 1 s, TE = 33 ms, multiband factor 4, FA = 59°, AM = 96 × 96, 48 axial slices, voxel size = 2.5 × 2.5 × 2.5 mm, no interslice gap). Standard preprocessing of the EPI data was performed, including motion‐correction, coregistration to each participant's reconstructed high‐resolution anatomical scan, smoothing with a three‐dimensional Gaussian kernel (FWHM = 3 mm), and intensity‐normalization. A general linear model (GLM) was calculated with regressors‐of‐interest for each stimulated location. The GLM also contained regressors‐of‐no‐interest for motion correction parameters and a linear scanner drift. The BOLD response was modeled with the SPM hemodynamic response function. Early visual areas V1 and V2 were defined by contrasting vertical vs. horizontal meridian stimulation conditions [69]. The mean ± SEM percentages V1 and V2 in the VOI gray matter across participants were as follows: V1: 80.6% ± 1.33% (min: 57.5%; max: 94.3%); V2: 19.3% ± 1.30% (min: 5.73%; max: 41.1%). Gray matter corresponding to other areas was negligible.

### 2.8 Statistical Analysis of Neurofeedback Training Results

To test whether participants achieved to increase or decrease Glx concentration with neurofeedback training, a linear fit was applied to percent changes in Glx concentration during induction relative to the reference concentration across all trials in each participant. Grubb's test was used to check for outliers in the fitted slopes; no outliers were identified. Slopes were compared to zero (corresponding to no change over trials) across participants in each group and experiment using a one‐sample Wilcoxon signed‐rank test. If participants achieved to increase Glx concentration through neurofeedback training, the slope would be positive. If participants achieved to decrease Glx concentration through neurofeedback training, the slope would be negative. Slopes were also compared between experimental and control groups using a Mann–Whitney *U* test. For this comparison, participants in the second experiment who had been in the control group in the first experiment (*n* = 2) were excluded, leaving 13 participants in the second experiment. The two‐tailed alpha‐level for each statistical test was set to 0.05. Effect sizes for the statistical tests are reported as *r*.

### 2.9 fMRS Control Analyses

A control analysis was performed to evaluate whether NAA + NAAG concentration used for Glx normalization remained stable over the course of neurofeedback training. To this end, a linear fit was applied to NAA + NAAG concentrations across induction trials for each participant. The slopes of these fits were compared to zero (corresponding to no change over induction trials) across participants in each group and experiment using a one‐sample Wilcoxon signed‐rank test. A similar analysis was performed on the composite concentration of Creatine and Phosphocreatine (Cr + PCr), which served as the normalization reference in another control analysis (see below).

For an exploratory post hoc analysis, Glx was normalized to Cr + PCr to assess whether similar neurofeedback training results were obtained. Note that this analysis is limited, as real‐time neurofeedback was not provided under this referencing scheme. The mean ± SEM CRLB of estimated Cr + PCr concentration across participants was 2.28% ± 0.05% (min: 2%; max: 4%) for fMRS reference scans and 2.98% ± 0.01% (min: 2%; max: 4%) for fMRS induction scans. For each participant, trial‐wise induced percent Glx concentration changes after Cr + PCr normalization within a session were examined for outlier values using the MAD criterion. In the first experiment, one outlier induction trial was removed from two participants in the experimental group and from one participant in the control group. In the second experiment, outlier induction trials were removed from three participants (four, one, and one trials, respectively). The statistical analysis of the Glx results followed the approach used in the primary analysis (see Section 2.8). Grubb's test revealed no outliers in the fitted slopes. To assess directional consistency with the primary analysis, slopes of linear fits to percent changes in Glx concentration normalized to Cr + PCr and NAA + NAAG concentrations were correlated across groups and experiments using Spearman's rank correlation analysis.

Finally, we also performed the primary analysis, in which Glx concentration was normalized to NAA + NAAG concentration, without Lorentzian linebroadening and without outlier trial removal as a control. The results corresponded to the original analysis used to calculate real‐time neurofeedback during training. Quality checks of the uncorrected fMRS data revealed the following mean ± SEM results across participants: CRLB across fMRS reference scans: 7.54% ± 0.07% (min: 6%; max: 10%) for Glx and 2.73% ± 0.05% (min: 2%; max: 4%) for NAA + NAAG. CRLB across fMRS induction scans: 8.05% ± 0.03% (min: 5%; max: 13%) for Glx and 3.53% ± 0.02% (min: 2%; max: 6%) for NAA + NAAG. SN ratio: 27.3 ± 0.34 (min: 17; max: 34) across fMRS reference scans and 17.2 ± 0.06 (min: 12; max: 24) across fMRS induction scans. FWHM: 4.99 ± 0.05 Hz (min: 4.07 Hz; max: 6.41 Hz) across fMRS reference scans and 5.01 ± 0.02 Hz (min: 3.57 Hz; max: 8.25 Hz) across fMRS induction scans.

## 3 Results

### 3.1 Strategies During Induction

Participant's strategies during induction, grouped into categories using generative AI (ChatGPT, Version 5.2; OpenAI, 2026), are shown in Table 1. See Table S2 for each participant's reported strategies. Similar strategies were employed by participants in previous fMRI‐based neurofeedback trainings (see [12, 45, 46]).

**TABLE 1:** Participants' strategies (grouped into categories) during induction. Note that participants could employ multiple strategies.

Thinking about	Experiment 1: Experimental Group	Experiment 1: Control Group	Experiment 2
Emotions, inner experience	*N* = 4	*N* = 5	*N* = 8
Social and autobiographical life	*N* = 9	*N* = 11	*N* = 4
Sports, games, and physical action	*N* = 5	*N* = 6	*N* = 7
Sensory, nature, and places	*N* = 7	*N* = 6	*N *= 7
Objects, activities, and knowledge	*N* = 9	*N* = 7	*N* = 9

### 3.2 Neurofeedback Training Results

**FIGURE 3:**
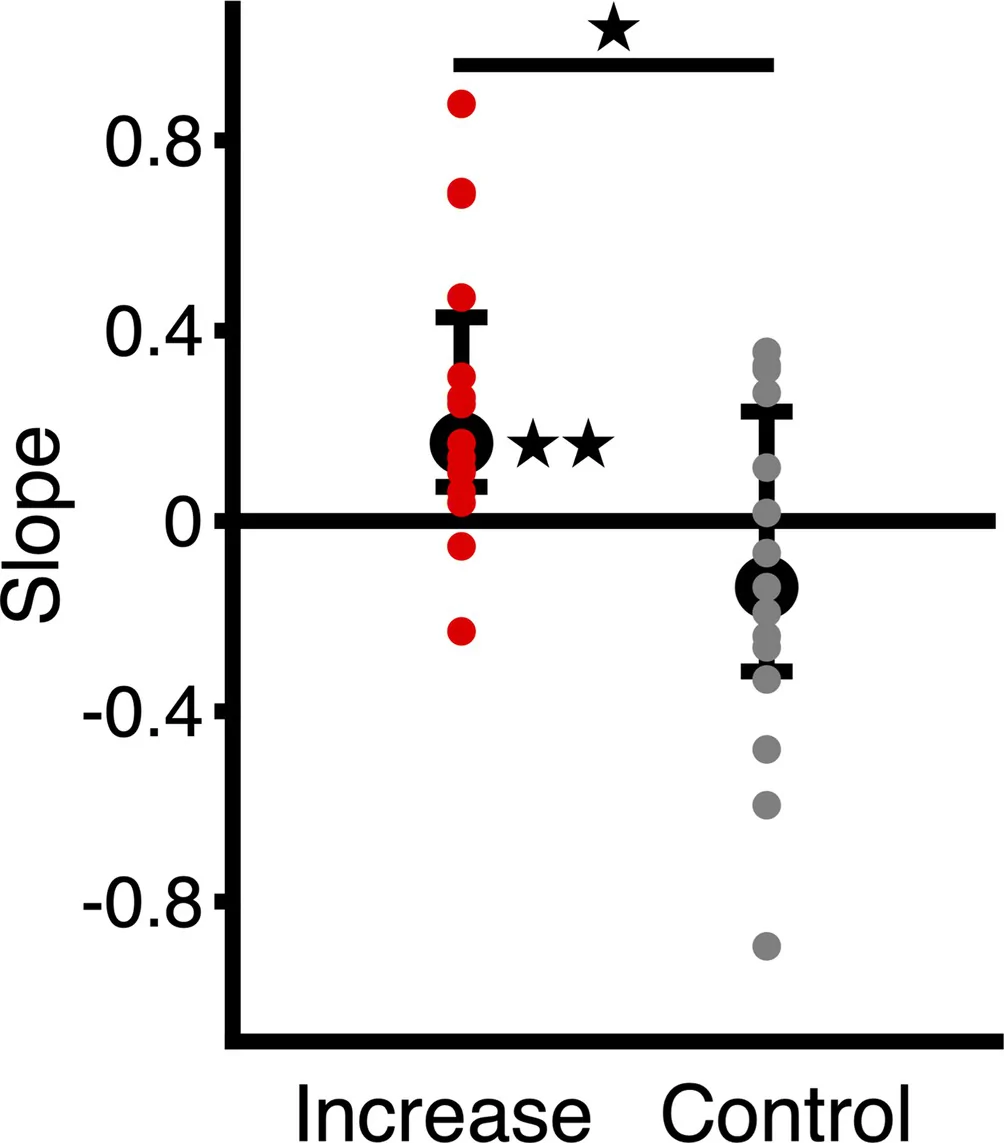
Neurofeedback training results in the first experiment. For each participant, a linear fit was performed on the trial‐wise change in Glx concentration during the induction phase relative to the reference concentration. Positive and negative slopes indicate an increase and decrease, respectively, in induced Glx concentration across trials. Zero corresponds to no change in concentration across trials. Large dots represent the median, with the interquartile range (IQR) shown across participants in the experimental (“Increase”) and control groups. Small dots show results for each participant. Slopes in the experimental group were significantly different from zero and significantly greater than in the control group (denoted by asterisks). * *p* < 0.05, ** *p* < 0.01.

**FIGURE 4:**
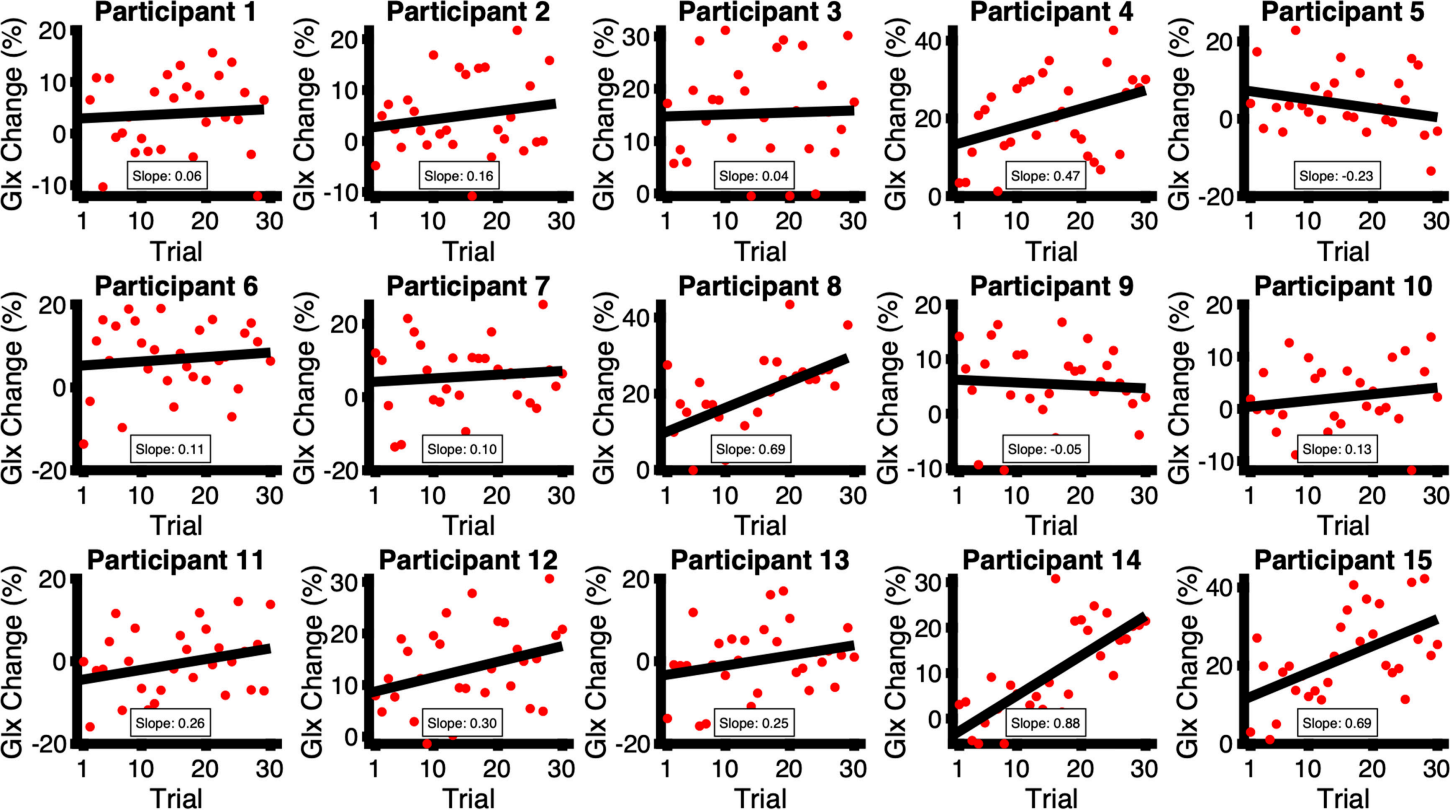
Trial‐by‐trial results in the experimental group in the first experiment. Each panel shows the trial‐wise induced percent change in Glx concentration relative to the reference concentration for a different participant. The line represents a linear fit across trials, and the slope of the fit, which was used for statistical testing, is indicated.

**FIGURE 5:**
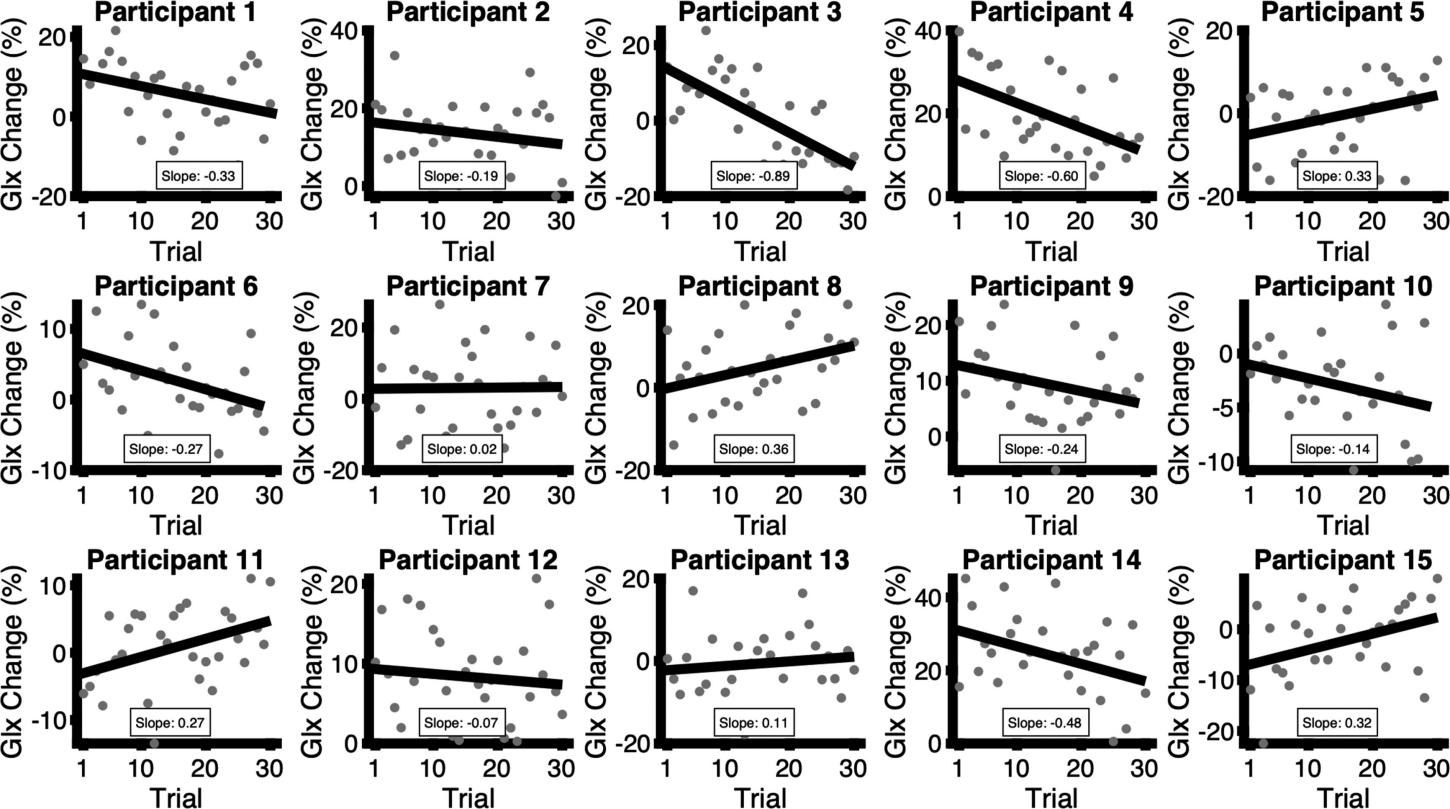
Trial‐by‐trial results in the control group in the first experiment. Otherwise same as Figure 4.

**FIGURE 6:**
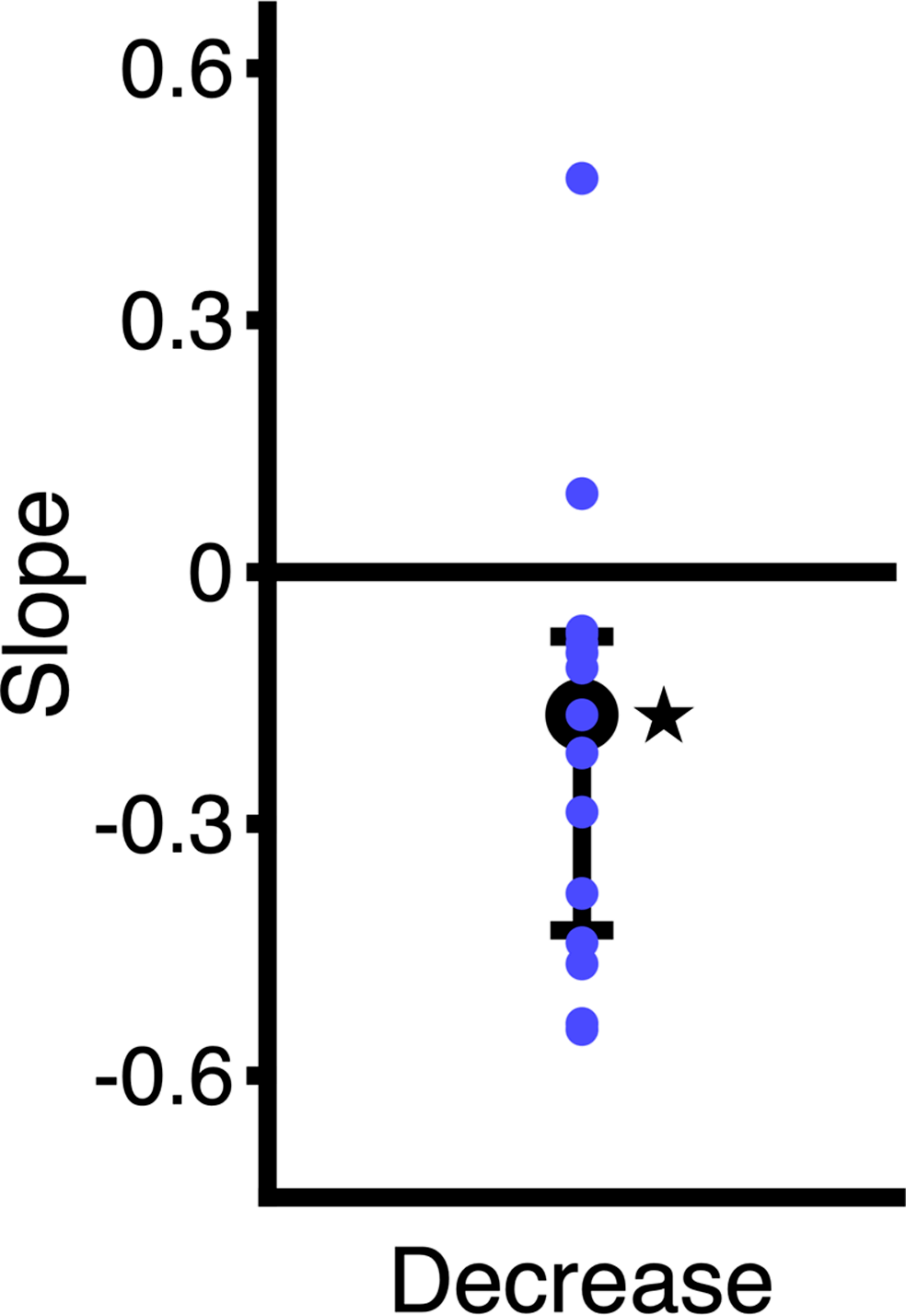
Neurofeedback training results in the second experiment. Otherwise same as Figure 3. * *p* < 0.05.

**FIGURE 7:**
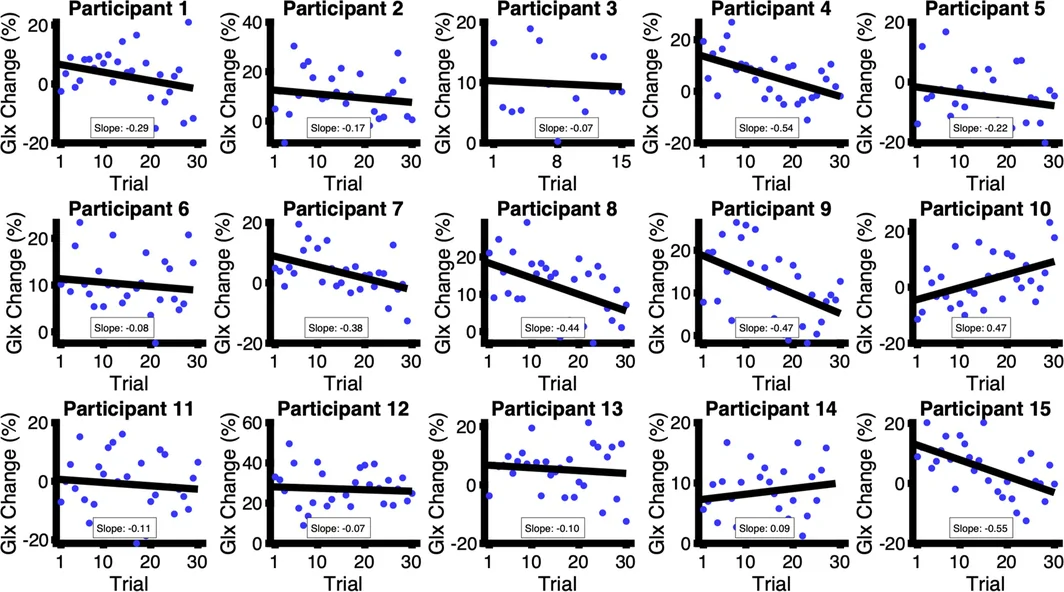
Trial‐by‐trial results in the second experiment. Otherwise same as Figure 4.

Figures 3, 4, 5 show fMRS results in the first experiment, in which the experimental group performed neurofeedback training to increase Glx concentration in the VOI, while the control group trained with random feedback. Slopes fitted to Glx concentration changes relative to the reference concentration across induction trials in the experimental group were significantly different from zero (Wilcoxon signed‐rank test; sample median = 0.16, *z* = 2.84, *p* = 0.005, *r* = 0.73), suggesting that participants learned to increase Glx concentration over the course of neurofeedback training (Figures 3 and 4). Slopes were not significantly different from zero in the control group (sample median = −0.14, *z* = −0.97, *p* = 0.33, *r* = −0.25) (Figures 3 and 5) and significantly greater in the experimental group than in the control group (Mann–Whitney *U* test; *U* = 54.0, *z* = 2.41, *p* = 0.02, *r* = 0.44) (Figure 3). These results suggest that the increase in Glx concentration in the experimental group was unlikely driven by performing the induction task irrespective of the veridicality of performance feedback or by random fluctuations of Glx concentrations over time. Figures 6 and 7 show fMRS results in the second experiment, in which participants performed neurofeedback training to decrease Glx concentration in the VOI. Slopes fitted to Glx concentration changes across induction trials were significantly different from zero (Wilcoxon signed‐rank test; sample median = −0.17, *z* = −2.44, *p* = 0.01, *r* = −0.63), suggesting that participants learned to decrease Glx concentration over the course of neurofeedback training. Slopes were not significantly different from those in the control group in the first experiment (Mann–Whitney *U* test; *U* = 85.0, *z* = −0.55, *p* = 0.58, *r* = −0.10). The mean ± SEM percentage Glx concentration change relative to the reference concentration across induction trials and participants was 8.83% ± 1.92% in the experimental group and 5.80% ± 2.06% in the control group in the first experiment, and 7.19% ± 1.88% in the second experiment.

### 3.3 fMRS Control Analyses

To evaluate whether control metabolites used for Glx normalization remained stable over the course of neurofeedback training, linear fits were performed to NAA + NAAG and Cr + PCr concentrations across induction trials. The slopes of these fits were not significantly different from zero in any experiment (Wilcoxon signed‐rank test; experimental group in first experiment: NAA + NAAG: sample median = 0.005, *z* = 0.80, *p* = 0.43, *r* = 0.21; Cr + PCr: sample median = −0.0004, *z* = −0.06, *p* = 0.95, *r* = −0.01; control group in first experiment: NAA + NAAG: sample median = −0.004, *z* = −1.70, *p* = 0.09, *r* = −0.44; Cr + PCr: sample median = 0.002, *z* = 1.42, *p* = 0.16, *r* = 0.37; second experiment: NAA + NAAG: sample median = −0.001, *z* = 0.16, *p* = 0.88, *r* = 0.04; Cr + PCr: sample median = 0.0003, *z* = −0.06, *p* = 0.95, *r* = −0.01). These results do not provide evidence of significant changes in control metabolites over the course of neurofeedback training.

For an exploratory post hoc analysis, we assessed whether similar neurofeedback training results were obtained when Glx concentration was normalized to Cr + PCr concentration. First, slopes of linear fits to percent changes in Glx concentration normalized to Cr + PCr and NAA + NAAG concentrations were correlated across groups and experiments using Spearman's rank correlation analysis. There was a significant positive correlation (*rho* = 0.57, *p* < 0.001), suggesting that Glx slopes obtained with different normalization approaches were overall trending in similar directions across groups and experiments. As in the primary analysis, slopes were compared with zero for each group and experiment using Wilcoxon signed‐rank tests. This analysis revealed similar results in the second experiment (slopes were significantly different from zero; sample median = −0.21, *z* = −2.10, *p* = 0.04, *r* = −0.54) and for the control group in the first experiment (slopes were not significantly different from zero; sample median = −0.07, *z* = −1.48, *p* = 0.14, *r* = −0.38) with no significant difference in slopes between both groups (Mann–Whitney *U* test; *U* = 80.0, *z* = −0.10, *p* = 0.92, *r* = −0.02). However, using Cr + PCr normalization, slopes in the experimental group in the first experiment were not significantly different from zero (sample median = −0.04, *z* = 0.11, *p* = 0.91, *r* = 0.03) and not significantly different from slopes in the control group (Mann–Whitney *U* test; *U* = 92.0, *z* = 0.83, *p* = 0.41, *r* = 0.15). These findings suggest that the training‐reference scheme influenced some effects, despite similar overall Glx changes with Cr + PCr and NAA + NAAG normalization.

Figures [Link], [Link], [Link] show fMRS results based on spectra without Lorentzian linebroadening and without metabolite concentration outlier removal. Slopes fitted to Glx concentration changes relative to the reference concentration across induction trials in the first experiment were significantly different from zero in the experimental group (Wilcoxon signed‐rank test; sample median = 0.17, *z* = 2.50, *p* = 0.01, *r* = 0.65), suggesting that participants learned to increase Glx concentration over the course of neurofeedback training (Figure S5). Slopes were not significantly different from zero in the control group (sample median = −0.04, *z* = −0.85, *p* = 0.39, *r* = −0.22) (Figure S6) and significantly greater in the experimental group than in the control group (Mann–Whitney *U* test; *U* = 53.0, *z* = 2.45, *p* = 0.01, *r* = 0.45). Slopes in the second experiment were significantly different from zero (Wilcoxon signed‐rank test; sample median = −0.11, *z* = −2.04, *p* = 0.04, *r* = −0.53), suggesting that participants learned to decrease Glx concentration over the course of neurofeedback training (Supplementary Figure S7). Slopes were not significantly different from those in the control group in the first experiment (Mann–Whitney *U* test; *U* = 84.0, *z* = −0.60, *p* = 0.55, *r* = −0.11). Overall, these results are consistent with those obtained after Lorentzian linebroadening and metabolite concentration outlier removal (see Section 3.2 and Figures 3, 4, 5, 6, 7). The mean ± SEM percentage Glx concentration change relative to the reference concentration across induction trials and participants was 9.57% ± 1.42% in the experimental group and 7.54% ± 1.25% in the control group in the first experiment, and 6.69% ± 1.32% in the second experiment.

## 4 Discussion

Here, we developed a neurofeedback training procedure using fMRS to modulate Glx concentration in the occipital lobes. Participants trained to increase or decrease Glx concentration and received real‐time feedback about their success or failure. The results showed that participants learned to induce Glx concentration changes in the desired direction over the course of neurofeedback training with veridical performance feedback. No significant changes in Glx concentration were found in a control group with random, that is, non‐informative, feedback.

In previous fMRI‐based neurofeedback trainings, participants learned to increase or decrease the BOLD signal in a region‐of‐interest or a network of regions [3, 4, 6]. Similarly, EEG‐based neurofeedback trainings showed that participants learned to induce increases or decreases of EEG band power [2, 70]. The present findings extend this work by demonstrating that fMRS‐based neurofeedback training can be used to increase or decrease excitatory neurotransmitter concentration within a VOI. All three approaches used to measure brain activity for neurofeedback training have distinct advantages and limitations. Compared with EEG, using fMRS, neurofeedback training effects may occur on a shorter training time scale [71], and the anatomical specificity is greater, albeit constrained by VOI size (see below). Relative to fMRI, fMRS provides lower anatomical specificity, but it offers the advantage of measuring neurotransmitter‐related processes. The three methods should be regarded as complementary, and their selection should be guided by the specific objectives of the neurofeedback training.

We used a 16‐s delay between induction and feedback on each trial. This delay followed previous designs in fMRI‐based neurofeedback training and was necessary to calculate the achieved change in Glx concentration and the corresponding feedback. In addition, this interval gave participants the opportunity to relax (i.e., let their minds wander in any direction as they wished) after the induction phase before feedback was presented. However, compared to the slow BOLD‐response in fMRI, the event‐related glutamatergic response occurs without much delay in fMRS [34]. Therefore, future fMRS‐based neurofeedback trainings should test whether the interval between induction and feedback can be reduced in order to present feedback about success or failure sooner after the end of the induction phase.

In the second experiment participants performed neurofeedback training to decrease Glx concentration. Previous fMRI‐based neurofeedback techniques reported that the BOLD signal can be reduced through training [72] but left the underlying neurochemical effects uncertain. Previous studies with fMRS found lower Glx concentrations during suppression induced by task‐conditions [59, 61, 73, 74] or transcranial stimulation [75, 76, 77]. The results of this study suggest that participants learned to actively induce a suppression in the VOI through neurofeedback training. This is supported by post‐scan reports (see Table S2), which showed that participants adopted active strategies during the induction phase in the second experiment (e.g., thinking about situations, memories, or emotions) rather than resting strategies such as “zooming‐out” or closing eyes. Of note, only some participants achieved to decrease Glx concentration below the reference concentration (see Figure 7). Where exactly the lower limit for the fMRS‐measured Glx concentration is and whether this differs between different brain regions is a question for future research. In this study involving healthy young participants, the reference value for Glx concentration was measured while participants relaxed (i.e., mind‐wandering without performing an explicit task) under minimal visual stimulation consisting of a centrally presented bull's eye on an otherwise blank screen. However, the value may have been different had participants performed an explicit task or been exposed to visual stimulation (e.g., checkerboard patterns) during the reference measurement (see [33, 62, 64, 78]) or in patient populations (see [79, 80, 81]).

An important question for interpreting fMRS results is what a change in Glx concentration means at the neuronal level [34, 82, 83]. In fMRS, Glx concentration is measured in a large VOI (2 × 2 × 2 cm in this study) and the change in signal is thought to be driven by several factors related to both neurotransmitter function and cellular metabolism, for example, a shift in glutamate concentrations across cytosol, vesicle, and extracellular compartments [34, 84]. However, the observed changes in Glx concentration during neurofeedback training may have been driven primarily by neurotransmission rather than metabolism for the following reasons. First, we used PRESS scans with long echo time (30 ms) to make our fMRS measurement sensitive to signals from glutamate in the synaptic cleft rather than glutamate stored in presynaptic vesicles [34, 82, 84]. Second, previous results showed that the event‐related glutamate response in fMRS occurs fast with little delay compared to the slow BOLD response in fMRI [34]. Therefore, we used a design with a short (16 s) induction phase to increase the probability that the measured glutamate concentration results from neurotransmission rather than slow metabolic processes following neuronal activity [85]. Third, in the control group with the same task but random feedback on success or failure during induction, there was no significant change in Glx concentration over time, even though participants tried to modulate the concentration (see Table S2 for post‐scan reports), resulting in the need for metabolic processes for glutamate reproduction after neurotransmission. This makes it unlikely that induced changes in Glx concentration over the course of neurofeedback training resulted primarily from metabolic processes rather than neurotransmission.

### 4.1 Limitations

Limitations of the current study should be considered in future research on fMRS‐based neurofeedback training. A first limitation is that BOLD linewidth narrowing effects were corrected only post hoc, rather than online during neurofeedback training. The rationale for omitting this correction during training was uncertainty about how this effect might manifest in a mind‐wandering task, in which the only difference between the reference and induction periods lies in the content of participants' thoughts. This paradigm differs substantially from fMRS paradigms examining neurotransmitter differences in the occipital lobes between clearly distinct visual stimulation conditions (e.g., [33, 62]). Consistent with this rationale, whether reference or induction spectra exhibited narrower linewidth varied considerably across participants. Moreover, similar neurofeedback training results were obtained with and without the correction. Nevertheless, future studies may benefit from a trial‐by‐trial evaluation of BOLD linewidth narrowing and the online application of the correction when warranted, in order to improve the specificity of neurofeedback training with respect to changes in neurotransmitter concentrations. Furthermore, future studies should consider conducting fMRS‐based neurofeedback training with other imaging sequences. Previous BOLD‐fMRI neurofeedback trainings using similar tasks and paradigms (e.g., [10]) found that participants can modulate their BOLD responses during mind‐wandering tasks. Therefore, with the present measurement sequence and experimental design, the possibility that participants' BOLD responses changed in parallel with the Glx modulation, cannot be fully excluded. In particular, during trials with large Glx increases, it is possible that participants' mental strategies also influenced BOLD responses alongside the learned Glx change. Future studies may benefit from using imaging sequences that allow a clearer separation of BOLD and Glx modulation in neurofeedback training using combined fMRI‐fMRS [33, 73, 86, 87, 88]. For example, a 3D EPI module could be integrated into a short‐TE MRS sequence, such as semi‐LASER, to enable concurrent fMRI and fMRS acquisition [33, 87].

A second limitation is that we did not perform online outlier trial removal or frequency/phase correction between the reference and induction spectra, apart from Siemens' internal processing of each spectrum prior to data export. The original rationale was to avoid potential delays introduced by the preprocessing steps. Future studies could consider implementing an explicitly defined criterion, such as excluding trials showing a Glx change above a pre‐determined percentage threshold, to remove outlier trials online. In addition, frequency/phase correction between the reference and induction spectra, when time permits, may help reduce confounding artifacts, such as motion.

A further limitation is that we only measured Glx concentration in a single VOI located in the occipital lobes. Our selection of VOI location was motivated by previous studies that successfully pioneered fMRI‐based neurofeedback training in early visual areas [10, 12, 28, 45, 46]. Moreover, a high SN ratio of the fMRS signal could be expected due to the proximity of the VOI to the coil elements and the high metabolism in the occipital lobes [60, 89]. Future studies should conduct neurofeedback training for VOIs in other parts of the brain to investigate whether modulation of Glx concentration is also possible there. In addition, due to the restriction to a single VOI, we were unable to measure whether neurofeedback training also led to modulations of Glx concentrations in other parts of the brain or whether the modulation occurred only in the occipital lobes. To answer this question, future studies should consider conducting neurofeedback training with multivoxel fMRS.

We did not observe a significant difference in fitted slopes between participants in the second experiment who trained to decrease Glx concentration and participants in the control group in the first experiment who received random feedback. The control group in the first experiment received approximately twice as much positive feedback (~50% of trials) as the decrease group (~23%). Thus, the control group in the first experiment may not have provided an equivalent comparison for the decrease group. Future studies could benefit from a new control group in which feedback probabilities are matched to those of the decrease group or in which feedback scores from the decrease group are played back to control participants (e.g., see [5]).

For neurofeedback training, Glx concentration was normalized to the composite measure of NAA and NAAG, a control metabolite concentration assumed to remain stable over time or conditions and therefore commonly used for normalization [59, 60, 74, 90, 91, 92]. Consistent with this assumption, NAA + NAAG concentration did not significantly change over the course of neurofeedback training. For an exploratory post hoc analysis, Glx concentration was normalized to the composite measure of Cr and PCr, another commonly used control metabolite concentration. The results showed similarities in the overall direction of normalized Glx concentration changes across groups and experiments. However, in the first experiment, a significant increase in Glx concentration in the experimental group was observed only with NAA + NAAG normalization. The extent to which normalization of Glx concentration influences neurofeedback training outcomes should be examined in future studies.

## 5 Conclusions

The results of this study suggest that real‐time neurofeedback training based on fMRS can be used to modulate Glx concentration in a VOI within a short period of time. This non‐invasive procedure, based on participants' own efforts and veridical feedback about their success or failure in inducing a Glx change, overcomes some of the limitations from previous fMRI‐based or EEG‐based neurofeedback techniques. Neurofeedback training procedures using fMRS may represent a new way to modulate neurotransmitter concentrations in healthy and clinical populations.

## Author Contributions

**Zhiyan Wang:** conceptualization, data curation, formal analysis, investigation, methodology, software, visualization, writing – original draft, writing – review and editing. **Savanna Babu:** writing – original draft, writing – review and editing. **Nina Beck:** investigation. **Sebastian M. Frank:** conceptualization, data curation, formal analysis, funding acquisition, investigation, methodology, project administration, resources, software, supervision, validation, visualization, writing – original draft, writing – review and editing.

## Funding

This research was supported by the Julitta und Richard Müller Stiftung and the Emmy Noether program of the Deutsche Forschungsgemeinschaft (DFG, German Research Foundation; 491290285).

## Conflicts of Interest

The authors declare no conflicts of interest.

## Supporting information


**Table S1:** fMRS checklist proposed by Choi et al. (2021).
**Table S2:** Participants' strategies during induction. Strategy category numbers correspond to the following: 1 = Emotions, inner experience; 2 = Social and autobiographical life; 3 = Sports, games, physical action; 4 = Sensory, nature, places; 5 = Objects, activities, knowledge.

**Figure S1:** PRESS spectra for induction trials in the experimental group in the first experiment. Spectra were corrected using Lorentzian linebroadening prior to LC model fitting. Each induction trial is indicated by the position on the y‐axis in ascending order, with the bottom line corresponding to the first induction trial. Each spectrum shows the mean across partcipants. The shaded area represents the standard‐error‐of‐the‐mean (SEM). Glx between 2.1 and 2.5 ppm and at 3.75 ppm. N‐acetyl‐aspartate at 2 ppm.

**Figure S2:** Spectra for induction trials in the control group in the first experiment. Otherwise same as Supplementary Figure 1.

**Figure S3:** Spectra for induction trials in the second experiment. Otherwise same as Supplementary Figure 1.

**Figure S4:** Difference spectra (induction minus reference) before (top row) and after correction (bottom row) using Lorentzian linebroadening. For each fMRS run and participant, the reference scan spectrum was subtracted from the mean spectrum across induction trials. The resulting difference spectra were first averaged across fMRS runs within each participant and subsequently across participants. The shaded area shows SEM. “Increase”: experimental group in the first experiment. “Control”: control group in the first experiment. “Decrease”: second experiment.

**Figure S5:** Trial‐by‐trial results in the experimental group in the first experiment without Lorentzian linebroadening and without metabolite concentration outlier removal. Otherwise same as Figure 4.

**Figure S6:** Trial‐by‐trial results in the control group in the first experiment. Otherwise same as Supplementary Figure 5.

**Figure S7:** Trial‐by‐trial results in the second experiment. Otherwise same as Supplementary Figure 5.

## Data Availability

The data that support the findings of this study are available from the corresponding author upon reasonable request.

## References

[1] R. C. deCharms , “Reading and Controlling Human Brain Activation Using Real‐Time Functional Magnetic Resonance Imaging,” Trends in Cognitive Sciences 11, no. 11 (2007): 473–481, 10.1016/j.tics.2007.08.014. 17988931

[2] R. Sitaram , T. Ros , L. Stoeckel , et al., “Closed‐Loop Brain Training: The Science of Neurofeedback,” Nature Reviews Neuroscience 18, no. 2 (2016): 86–100, 10.1038/nrn.2016.164. 28003656

[3] J. Sulzer , S. Haller , F. Scharnowski , et al., “Real‐Time fMRI Neurofeedback: Progress and Challenges,” NeuroImage 76 (2013): 386–399, 10.1016/j.neuroimage.2013.03.033. PMC4878436 23541800

[4] T. Watanabe , Y. Sasaki , K. Shibata , and M. Kawato , “Advances in fMRI Real‐Time Neurofeedback,” Trends in Cognitive Sciences xx (2017): 1–14, 10.1016/j.tics.2017.09.010. PMC5694350 29031663

[5] M. T. deBettencourt , J. D. Cohen , R. F. Lee , K. A. Norman , and N. B. Turk‐Browne , “Closed‐Loop Training of Attention With Real‐Time Brain Imaging,” Nature Neuroscience 18, no. 3 (2015): 470–475, 10.1038/nn.3940. PMC4503600 25664913

[6] R. C. deCharms , K. Christoff , G. H. Glover , J. M. Pauly , S. Whitfield , and J. D. E. Gabrieli , “Learned Regulation of Spatially Localized Brain Activation Using Real‐Time fMRI,” NeuroImage 21, no. 1 (2004): 436–443, 10.1016/j.neuroimage.2003.08.041. 14741680

[7] A. W. Keizer , R. S. Verment , and B. Hommel , “Enhancing Cognitive Control Through Neurofeedback: A Role of Gamma‐Band Activity in Managing Episodic Retrieval,” NeuroImage 49, no. 4 (2010): 3404–3413, 10.1016/j.neuroimage.2009.11.023. 19925870

[8] S. M. LaConte , “Decoding fMRI Brain States in Real‐Time,” NeuroImage 56, no. 2 (2011): 440–454, 10.1016/j.neuroimage.2010.06.052. 20600972

[9] T. Ros , J. Théberge , P. A. Frewen , et al., “Mind Over Chatter: Plastic Up‐Regulation of the fMRI Salience Network Directly After EEG Neurofeedback,” NeuroImage 65 (2013): 324–335, 10.1016/j.neuroimage.2012.09.046. PMC5051955 23022326

[10] F. Scharnowski , C. Hutton , O. Josephs , N. Weiskopf , and G. Rees , “Improving Visual Perception Through Neurofeedback,” Journal of Neuroscience 32, no. 49 (2012): 17830–17841, 10.1523/JNEUROSCI.6334-11.2012. PMC3520425 23223302

[11] D. Scheinost , T. W. Hsu , E. W. Avery , et al., “Connectome‐Based Neurofeedback: A Pilot Study to Improve Sustained Attention,” NeuroImage 212 (2020): 116684, 10.1016/j.neuroimage.2020.116684. PMC7165055 32114151

[12] K. Shibata , T. Watanabe , Y. Sasaki , and M. Kawato , “Perceptual Learning Incepted by Decoded fMRI Neurofeedback Without Stimulus Presentation,” Science 334, no. 6061 (2011): 1413–1415, 10.1126/science.1212003. PMC3297423 22158821

[13] N. Weiskopf , F. Scharnowski , R. Veit , R. Goebel , N. Birbaumer , and K. Mathiak , “Self‐Regulation of Local Brain Activity Using Real‐Time Functional Magnetic Resonance Imaging (fMRI),” Journal of Physiology, Paris 98, no. 4–6 (2004): 357–373, 10.1016/j.jphysparis.2005.09.019. 16289548

[14] J. D. Kropotov , V. A. Grin‐Yatsenko , V. A. Ponomarev , L. S. Chutko , E. A. Yakovenko , and I. S. Nikishena , “ERPs Correlates of EEG Relative Beta Training in ADHD Children,” International Journal of Psychophysiology 55, no. 1 (2005): 23–34, 10.1016/j.ijpsycho.2004.05.011. 15598513

[15] I. Moreno‐García , A. Cano‐Crespo , and F. Rivera , “Results of Neurofeedback in Treatment of Children With ADHD: A Systematic Review of Randomized Controlled Trials,” Applied Psychophysiology and Biofeedback 47, no. 3 (2022): 145–181, 10.1007/s10484-022-09547-1. 35612676

[16] A. Koizumi , K. Amano , A. Cortese , et al., “Fear Reduction Without Fear Through Reinforcement of Neural Activity That Bypasses Conscious Exposure,” Nature Human Behaviour 1, no. 1 (2016): 0006, 10.1038/s41562-016-0006. PMC5628620 28989977

[17] G. M. Russo , R. S. Balkin , and A. S. Lenz , “A Meta‐Analysis of Neurofeedback for Treating Anxiety‐Spectrum Disorders,” Journal of Counseling & Development 100, no. 3 (2022): 236–251, 10.1002/jcad.12424.

[18] A. Zilverstand , B. Sorger , P. Sarkheil , and R. Goebel , “fMRI Neurofeedback Facilitates Anxiety Regulation in Females With Spider Phobia,” Frontiers in Behavioral Neuroscience 9 (2015): 148, 10.3389/fnbeh.2015.00148. PMC4458693 26106309

[19] X. Li , Z. Li , Z. Zou , et al., “Real‐Time fMRI Neurofeedback Training Changes Brain Degree Centrality and Improves Sleep in Chronic Insomnia Disorder: A Resting‐State fMRI Study,” Frontiers in Molecular Neuroscience 15 (2022): 825286, 10.3389/fnmol.2022.825286. PMC8904428 35283729

[20] J. I. Recio‐Rodriguez , M. Fernandez‐Crespo , N. Sanchez‐Aguadero , et al., “Neurofeedback to Enhance Sleep Quality and Insomnia: A Systematic Review and Meta‐Analysis of Randomized Clinical Trials,” Frontiers in Neuroscience 18 (2024): 1450163, 10.3389/fnins.2024.1450163. PMC11576419 39568666

[21] H. Chapin , E. Bagarinao , and S. Mackey , “Real‐Time fMRI Applied to Pain Management,” Neuroscience Letters 520, no. 2 (2012): 174–181, 10.1016/j.neulet.2012.02.076. PMC3377818 22414861

[22] R. C. deCharms , F. Maeda , G. H. Glover , et al., “Control Over Brain Activation and Pain Learned by Using Real‐Time Functional MRI,” Proceedings of the National Academy of Sciences 102, no. 51 (2005): 18626–18631, 10.1073/pnas.0505210102. PMC1311906 16352728

[23] K. Emmert , M. Breimhorst , T. Bauermann , et al., “Active Pain Coping Is Associated With the Response in Real‐Time fMRI Neurofeedback During Pain,” Brain Imaging and Behavior 11, no. 3 (2017): 712–721, 10.1007/s11682-016-9547-0. PMC5486591 27071949

[24] T. L. Kvamme , T. Ros , and M. Overgaard , “Can Neurofeedback Provide Evidence of Direct Brain‐Behavior Causality?,” NeuroImage 258 (2022): 119400, 10.1016/j.neuroimage.2022.119400. 35728786

[25] J. J. McGough , “Neurofeedback for ADHD: Time to Call It Quits?,” American Journal of Psychiatry 179, no. 12 (2022): 888–889, 10.1176/appi.ajp.20220861. 36453036

[26] M. Schabus , H. Griessenberger , M.‐T. Gnjezda , D. P. J. Heib , M. Wislowska , and K. Hoedlmoser , “Better Than Sham? A Double‐Blind Placebo‐Controlled Neurofeedback Study in Primary Insomnia,” Brain 140, no. 4 (2017): 1041–1052, 10.1093/brain/awx011. PMC5382955 28335000

[27] H. Marzbani , H. Marateb , and M. Mansourian , “Methodological Note: Neurofeedback: A Comprehensive Review on System Design, Methodology and Clinical Applications,” Basic and Clinical Neuroscience Journal 7, no. 2 (2016): 143–158, 10.15412/J.BCN.03070208. PMC4892319 27303609

[28] Z. Wang , T. Watanabe , and Y. Sasaki , “fMRI Neurofeedback for Perception and Attention,” in fMRI Neurofeedback (Elsevier, 2021), 85–105, 10.1016/B978-0-12-822421-2.00010-7.

[29] M. Hampson and D. Linden , “Protocol Design in fMRI Neurofeedback Studies,” in fMRI Neurofeedback (Elsevier, 2021), 57–79, 10.1016/B978-0-12-822421-2.00012-0.

[30] N. Weiskopf , K. Mathiak , S. W. Bock , et al., “Principles of a Brain‐Computer Interface (BCI) Based on Real‐Time Functional Magnetic Resonance Imaging (fMRI),” IEEE Transactions on Bio‐Medical Engineering 51, no. 6 (2004): 966–970, 10.1109/TBME.2004.827063. 15188865

[31] N. Weiskopf , R. Sitaram , O. Josephs , et al., “Real‐Time Functional Magnetic Resonance Imaging: Methods and Applications,” Magnetic Resonance Imaging 25, no. 6 (2007): 989–1003, 10.1016/j.mri.2007.02.007. 17451904

[32] I. B. Ip and H. Bridge , “Investigating the Neurochemistry of the Human Visual System Using Magnetic Resonance Spectroscopy,” Brain Structure and Function 227, no. 4 (2022): 1491–1505, 10.1007/s00429-021-02273-0. PMC9046312 33900453

[33] I. B. Ip , U. E. Emir , A. J. Parker , J. Campbell , and H. Bridge , “Comparison of Neurochemical and BOLD Signal Contrast Response Functions in the Human Visual Cortex,” Journal of Neuroscience 39, no. 40 (2019): 7968–7975, 10.1523/JNEUROSCI.3021-18.2019. PMC6774413 31358655

[34] P. G. Mullins , “Towards a Theory of Functional Magnetic Resonance Spectroscopy (fmrs): A Meta‐Analysis and Discussion of Using mrs to Measure Changes in Neurotransmitters in Real Time,” Scandinavian Journal of Psychology 59, no. 1 (2018): 91–103, 10.1111/sjop.12411. 29356002

[35] P. G. Mullins , L. M. Rowland , R. E. Jung , and W. L. Sibbitt , “A Novel Technique to Study the Brain's Response to Pain: Proton Magnetic Resonance Spectroscopy,” NeuroImage 26, no. 2 (2005): 642–646, 10.1016/j.neuroimage.2005.02.001. 15907322

[36] D. Pasanta , J. L. He , T. Ford , G. Oeltzschner , D. J. Lythgoe , and N. A. Puts , “Functional MRS Studies of GABA and Glutamate/Glx—A Systematic Review and Meta‐Analysis,” Neuroscience and Biobehavioral Reviews 144 (2023): 104940, 10.1016/j.neubiorev.2022.104940. PMC9846867 36332780

[37] J. A. Stanley and N. Raz , “Functional Magnetic Resonance Spectroscopy: The “New” MRS for Cognitive Neuroscience and Psychiatry Research,” Frontiers in Psychiatry 9 (2018): 76, 10.3389/fpsyt.2018.00076. PMC5857528 29593585

[38] P. G. Antuono , J. L. Jones , Y. Wang , and S.‐J. Li , “Decreased Glutamate + Glutamine in Alzheimer's Disease Detected In Vivo With 1 H‐MRS at 0.5 T,” Neurology 56, no. 6 (2001): 737–742, 10.1212/WNL.56.6.737. 11274307

[39] L. Velu , L. Pellerin , A. Julian , et al., “Early Rise of Glutamate‐Glutamine Levels in Mild Cognitive Impairment: Evidence for Emerging Excitotoxicity,” Journal of Neuroradiology 51, no. 2 (2024): 168–175, 10.1016/j.neurad.2023.09.003. 37777087

[40] D. G. Kondo , T. L. Hellem , Y.‐H. Sung , et al., “Review: Magnetic Resonance Spectroscopy Studies of Pediatric Major Depressive Disorder,” Depression Research and Treatment 2011 (2011): 1–13, 10.1155/2011/650450. PMC3003951 21197097

[41] G. L. Sarlo and K. F. Holton , “Brain Concentrations of Glutamate and GABA in Human Epilepsy: A Review,” Seizure 91 (2021): 213–227, 10.1016/j.seizure.2021.06.028. 34233236

[42] J. Kumar , E. B. Liddle , C. C. Fernandes , et al., “Glutathione and Glutamate in Schizophrenia: A 7T MRS Study,” Molecular Psychiatry 25, no. 4 (2020): 873–882, 10.1038/s41380-018-0104-7. PMC7156342 29934548

[43] K. Merritt , A. Egerton , M. J. Kempton , M. J. Taylor , and P. K. McGuire , “Nature of Glutamate Alterations in Schizophrenia,” JAMA Psychiatry 73, no. 7 (2016): 665, 10.1001/jamapsychiatry.2016.0442. 27304221

[44] Y. Koush , M. A. Elliott , and K. Mathiak , “Single Voxel Proton Spectroscopy for Neurofeedback at 7 Tesla,” Materials 4, no. 9 (2011): 1548–1563, 10.3390/ma4091548. PMC3886242 24416473

[45] Z. Wang , M. Tamaki , S. M. Frank , et al., “Visual Perceptual Learning of a Primitive Feature in Human V1/V2 as a Result of Unconscious Processing, Revealed by Decoded Functional MRI Neurofeedback (DecNef),” Journal of Vision 21, no. 8 (2021): 24, 10.1167/jov.21.8.24. PMC8399321 34431964

[46] K. Amano , K. Shibata , M. Kawato , Y. Sasaki , and T. Watanabe , “Learning to Associate Orientation With Color in Early Visual Areas by Associative Decoded fMRI Neurofeedback,” Current Biology: CB 26, no. 14 (2016): 1861–1866, 10.1016/j.cub.2016.05.014. PMC4961545 27374335

[47] I. Choi , O. C. Andronesi , P. Barker , et al., “Spectral Editing in1 H Magnetic Resonance Spectroscopy: Experts' Consensus Recommendations,” NMR in Biomedicine 34, no. 5 (2021): e4411, 10.1002/nbm.4411. PMC8557623 32946145

[48] P. A. Bottomley , “Spatial Localization in NMR Spectroscopy in Vivo,” Annals of the New York Academy of Sciences 508, no. 1 (1987): 333–348. Portico, 10.1111/j.1749-6632.1987.tb32915.x. 3326459

[49] P. A. Bottomley , “NMR in Medicine,” Computerized Radiology 8, no. 2 (1984): 57–77, 10.1016/0730-4862(84)90065-9. 6373119

[50] R. J. Ogg , P. B. Kingsley , and J. S. Taylor , “WET, a T1‐ and B1‐Insensitive Water‐Suppression Method for In Vivo Localized 1H NMR Spectroscopy,” Journal of Magnetic Resonance. Series B 104, no. 1 (1994): 1–10, 10.1006/jmrb.1994.1048. 8025810

[51] S. M. Frank , M. Becker , A. Qi , et al., “Efficient Learning in Children With Rapid GABA Boosting During and After Training,” Current Biology 32, no. 23 (2022): 5022–5030.e7, 10.1016/j.cub.2022.10.021. 36384138

[52] M. Y. Mel'nikov , D. D. Bezmaternykh , A. A. Savelov , et al., “Real‐Time fMRI Neurofeedback Compared to Cognitive Behavioral Therapy in a Pilot Study for the Treatment of Mild and Moderate Depression,” European Archives of Psychiatry and Clinical Neuroscience 273, no. 5 (2023): 1139–1149, 10.1007/s00406-022-01462-0. PMC10359372 35908116

[53] B. Sorger , F. Scharnowski , D. E. J. Linden , M. Hampson , and K. D. Young , “Control Freaks: Towards Optimal Selection of Control Conditions for fMRI Neurofeedback Studies,” NeuroImage 186 (2019): 256–265, 10.1016/j.neuroimage.2018.11.004. PMC6338498 30423429

[54] D. H. Brainard , “The Psychophysics Toolbox,” Spatial Vision 10, no. 4 (1997): 433–436. 9176952

[55] D. G. Pelli , “The VideoToolbox Software for Visual Psychophysics: Transforming Numbers Into Movies,” Spatial Vision 10, no. 4 (1997): 437–442. 9176953

[56] G. Oeltzschner , H. J. Zöllner , S. C. N. Hui , et al., “Osprey: Open‐Source Processing, Reconstruction & Estimation of Magnetic Resonance Spectroscopy Data,” Journal of Neuroscience Methods 343 (2020): 108827, 10.1016/j.jneumeth.2020.108827. PMC7477913 32603810

[57] S. W. Provencher , “Estimation of Metabolite Concentrations From Localized In Vivo Proton NMR Spectra,” Magnetic Resonance in Medicine 30, no. 6 (1993): 672–679, 10.1002/mrm.1910300604. 8139448

[58] S. W. Provencher , “Automatic Quantitation of Localized In Vivo 1 H Spectra With LCModel,” NMR in Biomedicine 14, no. 4 (2001): 260–264, 10.1002/nbm.698. 11410943

[59] M. Becker , S. Babu , Z. Wang , and S. M. Frank , “Suppression of Task‐Irrelevant Learning Achieved Through Withdrawal of Excitation in Early Sensory Areas,” Cerebral Cortex 35, no. 12 (2025): bhaf333, 10.1093/cercor/bhaf333. 41396833

[60] S. M. Frank , M. Becker , W. M. Malloni , Y. Sasaki , M. W. Greenlee , and T. Watanabe , “Protocol to Conduct Functional Magnetic Resonance Spectroscopy in Different Age Groups of Human Participants,” STAR Protocols 4, no. 3 (2023): 102493, 10.1016/j.xpro.2023.102493. PMC10448431 37572324

[61] S. M. Frank , L. Forster , M. Pawellek , et al., “Visual Attention Modulates Glutamate‐Glutamine Levels in Vestibular Cortex: Evidence From Magnetic Resonance Spectroscopy,” Journal of Neuroscience: The Official Journal of the Society for Neuroscience 41, no. 9 (2021): 1970–1981, 10.1523/JNEUROSCI.2018-20.2020. PMC7939083 33452222

[62] P. Bednařík , I. Tkáč , F. Giove , et al., “Neurochemical and BOLD Responses During Neuronal Activation Measured in the Human Visual Cortex at 7 Tesla,” Journal of Cerebral Blood Flow & Metabolism 35, no. 4 (2015): 601–610, 10.1038/jcbfm.2014.233. PMC4420878 25564236

[63] N. Just , “Validity and Specificity of BOLD Effects and Their Correction in 1H‐fMRS,” Frontiers in Neuroscience 18 (2024): 1433468, 10.3389/fnins.2024.1433468. PMC11438475 39347531

[64] Y. Koush , D. L. Rothman , K. L. Behar , R. A. De Graaf , and F. Hyder , “Human Brain Functional MRS Reveals Interplay of Metabolites Implicated in Neurotransmission and Neuroenergetics,” Journal of Cerebral Blood Flow & Metabolism 42, no. 6 (2022): 911–934, 10.1177/0271678X221076570. PMC9125492 35078383

[65] M. Wilson , S. M. Finney , and W. T. Clarke , “The Impact of BOLD Induced Linewidth Modulation on Functional1 H MRS Analysis,” bioRxiv. Preprint posted online March 6, 2026. 10.64898/2026.03.06.710034. PMC13591006 42764503

[66] A. M. Dale , B. Fischl , and M. I. Sereno , “Cortical Surface‐Based Analysis. I. Segmentation and Surface Reconstruction,” NeuroImage 9, no. 2 (1999): 179–194, 10.1006/nimg.1998.0395. 9931268

[67] B. Fischl , M. I. Sereno , and A. M. Dale , “Cortical Surface‐Based Analysis. II: Inflation, Flattening, and a Surface‐Based Coordinate System,” NeuroImage 9, no. 2 (1999): 195–207, 10.1006/nimg.1998.0396. 9931269

[68] A. L. Beer , T. Watanabe , R. Ni , Y. Sasaki , and G. J. Andersen , “3D Surface Perception From Motion Involves a Temporal–Parietal Network,” European Journal of Neuroscience 30, no. 4 (2009): 703–713, 10.1111/j.1460-9568.2009.06857.x. PMC2902171 19674088

[69] E. A. DeYoe , G. J. Carman , P. Bandettini , et al., “Mapping Striate and Extrastriate Visual Areas in Human Cerebral Cortex,” Proceedings of the National Academy of Sciences of the United States of America 93, no. 6 (1996): 2382–2386, 10.1073/pnas.93.6.2382. PMC39805 8637882

[70] T. M. Sokhadze , R. L. Cannon , and D. L. Trudeau , “EEG Biofeedback as a Treatment for Substance Use Disorders: Review, Rating of Efficacy, and Recommendations for Further Research,” Applied Psychophysiology and Biofeedback 33, no. 1 (2008): 1–28, 10.1007/s10484-007-9047-5. PMC2259255 18214670

[71] C. Loriette , C. Ziane , and S. Ben Hamed , “Neurofeedback for Cognitive Enhancement and Intervention and Brain Plasticity,” Revue Neurologique 177, no. 9 (2021): 1133–1144, 10.1016/j.neurol.2021.08.004. 34674879

[72] K. D. Young , V. Zotev , R. Phillips , et al., “Real‐Time fMRI Neurofeedback Training of Amygdala Activity in Patients With Major Depressive Disorder,” PLoS ONE 9, no. 2 (2014): e88785, 10.1371/journal.pone.0088785. PMC3921228 24523939

[73] D. Apšvalka , A. Gadie , M. Clemence , and P. G. Mullins , “Event‐Related Dynamics of Glutamate and BOLD Effects Measured Using Functional Magnetic Resonance Spectroscopy (fMRS) at 3T in a Repetition Suppression Paradigm,” NeuroImage 118 (2015): 292–300, 10.1016/j.neuroimage.2015.06.015. 26072254

[74] N. Lally , P. G. Mullins , M. V. Roberts , D. Price , T. Gruber , and C. Haenschel , “Glutamatergic Correlates of Gamma‐Band Oscillatory Activity During Cognition: A Concurrent ER‐MRS and EEG Study,” NeuroImage 85, no. Pt 2 (2014): 823–833, 10.1016/j.neuroimage.2013.07.049. 23891885

[75] N. Michael , M. Gösling , M. Reutemann , et al., “Metabolic Changes After Repetitive Transcranial Magnetic Stimulation (rTMS) of the Left Prefrontal Cortex: A Sham‐Controlled Proton Magnetic Resonance Spectroscopy (^1^H MRS) Study of Healthy Brain,” European Journal of Neuroscience 17, no. 11 (2003): 2462–2468, 10.1046/j.1460-9568.2003.02683.x. 12814378

[76] I. Moxon‐Emre , Z. J. Daskalakis , D. M. Blumberger , et al., “Modulation of Dorsolateral Prefrontal Cortex Glutamate/Glutamine Levels Following Repetitive Transcranial Magnetic Stimulation in Young Adults With Autism,” Frontiers in Neuroscience 15 (2021): 711542, 10.3389/fnins.2021.711542. PMC8527173 34690671

[77] C. J. Stagg , J. G. Best , M. C. Stephenson , et al., “Polarity‐Sensitive Modulation of Cortical Neurotransmitters by Transcranial Stimulation,” Journal of Neuroscience 29, no. 16 (2009): 5202–5206, 10.1523/JNEUROSCI.4432-08.2009. PMC6665468 19386916

[78] K. Kurcyus , E. Annac , N. M. Hanning , et al., “Opposite Dynamics of GABA and Glutamate Levels in the Occipital Cortex During Visual Processing,” Journal of Neuroscience 38, no. 46 (2018): 9967–9976, 10.1523/JNEUROSCI.1214-18.2018. PMC6234295 30282724

[79] J. J. Luykx , K. G. Laban , M. P. van den Heuvel , et al., “Region and State Specific Glutamate Downregulation in Major Depressive Disorder: A Meta‐Analysis of (1)H‐MRS Findings,” Neuroscience and Biobehavioral Reviews 36, no. 1 (2012): 198–205, 10.1016/j.neubiorev.2011.05.014. 21672551

[80] A. Marsman , M. P. van den Heuvel , D. W. J. Klomp , R. S. Kahn , P. R. Luijten , and H. E. Hulshoff Pol , “Glutamate in Schizophrenia: A Focused Review and Meta‐Analysis of ^1^H‐MRS Studies,” Schizophrenia Bulletin 39, no. 1 (2013): 120–129, 10.1093/schbul/sbr069. PMC3523901 21746807

[81] S. Ramadan , A. Lin , and P. Stanwell , “Glutamate and Glutamine: A Review of In Vivo MRS in the Human Brain,” NMR in Biomedicine 26, no. 12 (2013): 1630–1646, 10.1002/nbm.3045. PMC3849600 24123328

[82] R. A. Kauppinen , T. R. Pirttilä , S. O. Auriola , and S. R. Williams , “Compartmentation of Cerebral Glutamate In Situ as Detected by 1H/13C N.M.R,” Biochemical Journal 298, no. Pt 1 (1994): 121–127, 10.1042/bj2980121. PMC1137991 7907470

[83] C. J. Stagg , V. Bachtiar , and H. Johansen‐Berg , “What Are We Measuring With GABA Magnetic Resonance Spectroscopy?,” Communicative & Integrative Biology 4, no. 5 (2011): 573–575, 10.4161/cib.16213. PMC3204132 22046466

[84] C. A. Lea‐Carnall , W. El‐Deredy , C. J. Stagg , S. R. Williams , and N. J. Trujillo‐Barreto , “A Mean‐Field Model of Glutamate and GABA Synaptic Dynamics for Functional MRS,” NeuroImage 266 (2023): 119813, 10.1016/j.neuroimage.2022.119813. PMC7614487 36528313

[85] R. S. Koolschijn , W. T. Clarke , I. B. Ip , U. E. Emir , and H. C. Barron , “Event‐Related Functional Magnetic Resonance Spectroscopy,” NeuroImage 276 (2023): 120194, 10.1016/j.neuroimage.2023.120194. PMC7614684 37244321

[86] J. Dorst , T. Borbath , K. Landheer , N. Avdievich , and A. Henning , “Simultaneous Detection of Metabolite Concentration Changes, Water BOLD Signal and pH Changes During Visual Stimulation in the Human Brain at 9.4T,” Journal of Cerebral Blood Flow and Metabolism: Official Journal of the International Society of Cerebral Blood Flow and Metabolism 42, no. 6 (2022): 1104–1119, 10.1177/0271678X221075892. PMC9121534 35060409

[87] I. B. Ip , A. Berrington , A. T. Hess , A. J. Parker , U. E. Emir , and H. Bridge , “Combined fMRI‐MRS Acquires Simultaneous Glutamate and BOLD‐fMRI Signals in the Human Brain,” NeuroImage 155 (2017): 113–119, 10.1016/j.neuroimage.2017.04.030. PMC5519502 28433623

[88] S. Posse , B. Sa De La Rocque Guimaraes , T. Hutchins‐Delgado , et al., “On the Acquisition of the Water Signal During Water Suppression: High‐Speed MR Spectroscopic Imaging With Water Referencing and Concurrent Functional MRI,” NMR in Biomedicine 34, no. 5 (2021): e4261, 10.1002/nbm.4261. PMC7390701 31999397

[89] N. A. J. Puts and R. A. E. Edden , “In Vivo Magnetic Resonance Spectroscopy of GABA: A Methodological Review,” Progress in Nuclear Magnetic Resonance Spectroscopy 60 (2012): 29–41, 10.1016/j.pnmrs.2011.06.001. PMC3383792 22293397

[90] P. G. Mullins , D. J. McGonigle , R. L. O'Gorman , et al., “Current Practice in the Use of MEGA‐PRESS Spectroscopy for the Detection of GABA,” NeuroImage 86 (2014): 43–52, 10.1016/j.neuroimage.2012.12.004. PMC3825742 23246994

[91] P. J. Pouwels and J. Frahm , “Regional Metabolite Concentrations in Human Brain as Determined by Quantitative Localized Proton MRS,” Magnetic Resonance in Medicine 39, no. 1 (1998): 53–60, 10.1002/mrm.1910390110. 9438437

[92] Z. Wang , S. Wiborg , A. Wittmann , N. Beck , S. Hirschle , D. Aschenbrenner , M. Becker , and S. M. Frank , “Goal‐Directed Visual Information Processing with GABAergic Inhibition in Parietal Cortex,” Elife 15 (2026): RP109344, 10.7554/elife.109344.1.

