# Supplementary material for: Real‐Time Neurofeedback Training to Modulate Glutamate‐Glutamine Concentration Using Functional Magnetic Resonance Spectroscopy
Source: NMR Biomed. 2026 Sep 21;39(11):e70399. doi: 10.1002/nbm.70399 (PMC13593363; doi:10.1002/nbm.70399)
Supplement: Supplementary file 2 [file NBM-39-e70399-s002.pdf]

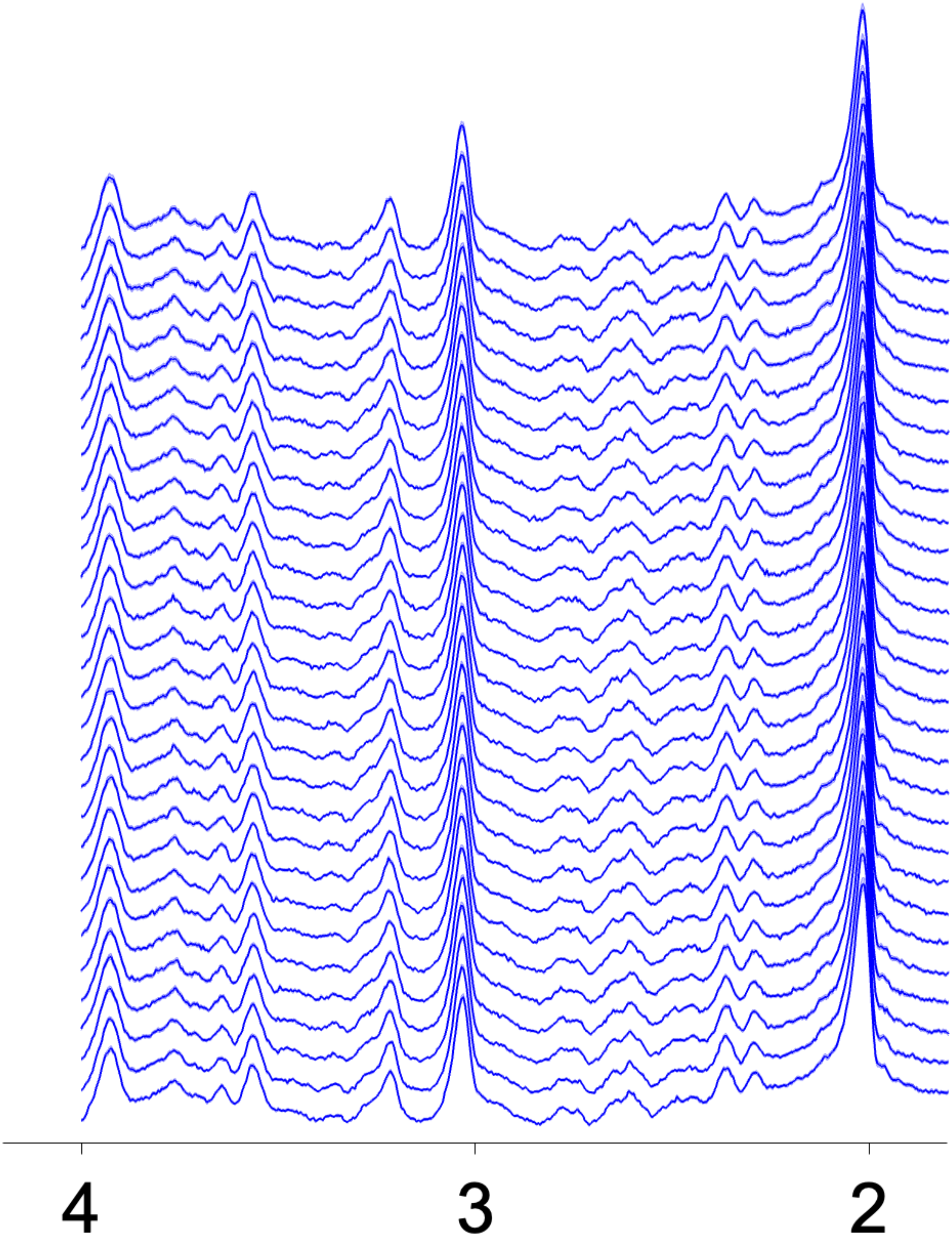
4
3
2
Frequency (ppm)
