# Supplementary material for: Real‐Time Neurofeedback Training to Modulate Glutamate‐Glutamine Concentration Using Functional Magnetic Resonance Spectroscopy
Source: NMR Biomed. 2026 Sep 21;39(11):e70399. doi: 10.1002/nbm.70399 (PMC13593363; doi:10.1002/nbm.70399)
Supplement: Supplementary file 3 [file NBM-39-e70399-s003.pdf]

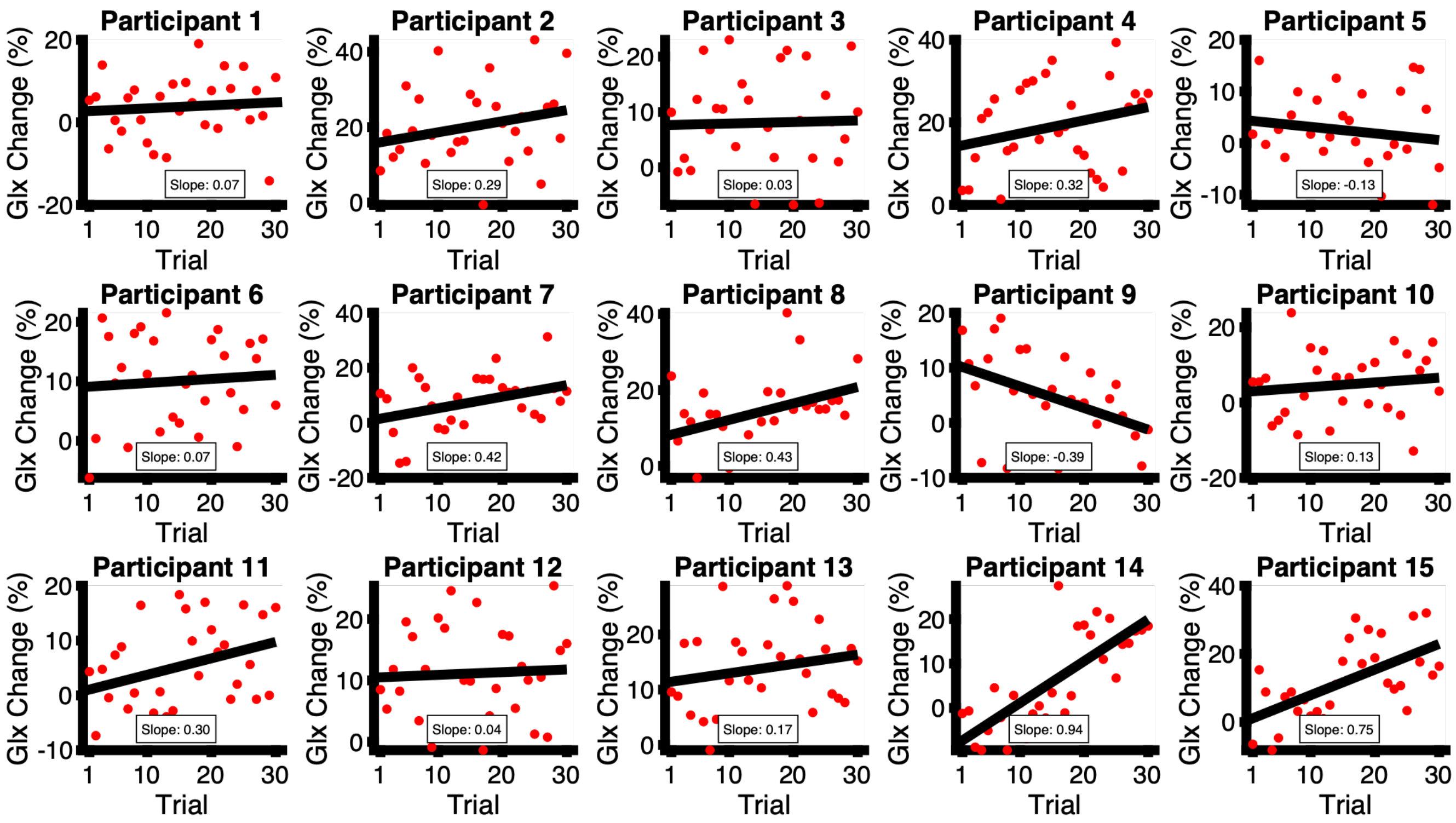

Participant 1
Slope: 0.07
Participant 2
Slope: 0.29
Participant 3
Slope: 0.03
Participant 4
Slope: 0.32
Participant 5
Slope: -0.13
Participant 6
Slope: 0.07
Participant 7
Slope: 0.42
Participant 8
Slope: 0.43
Participant 9
Slope: -0.39
Participant 10
Slope: 0.13
Participant 11
Slope: 0.30
Participant 12
Slope: 0.04
Participant 13
Slope: 0.17
Participant 14
Slope: 0.94
Participant 15
Slope: 0.75
Glx Change (%)
Trial
