# Supplementary material for: Real‐Time Neurofeedback Training to Modulate Glutamate‐Glutamine Concentration Using Functional Magnetic Resonance Spectroscopy
Source: NMR Biomed. 2026 Sep 21;39(11):e70399. doi: 10.1002/nbm.70399 (PMC13593363; doi:10.1002/nbm.70399)
Supplement: Supplementary file 4 [file NBM-39-e70399-s004.pdf]

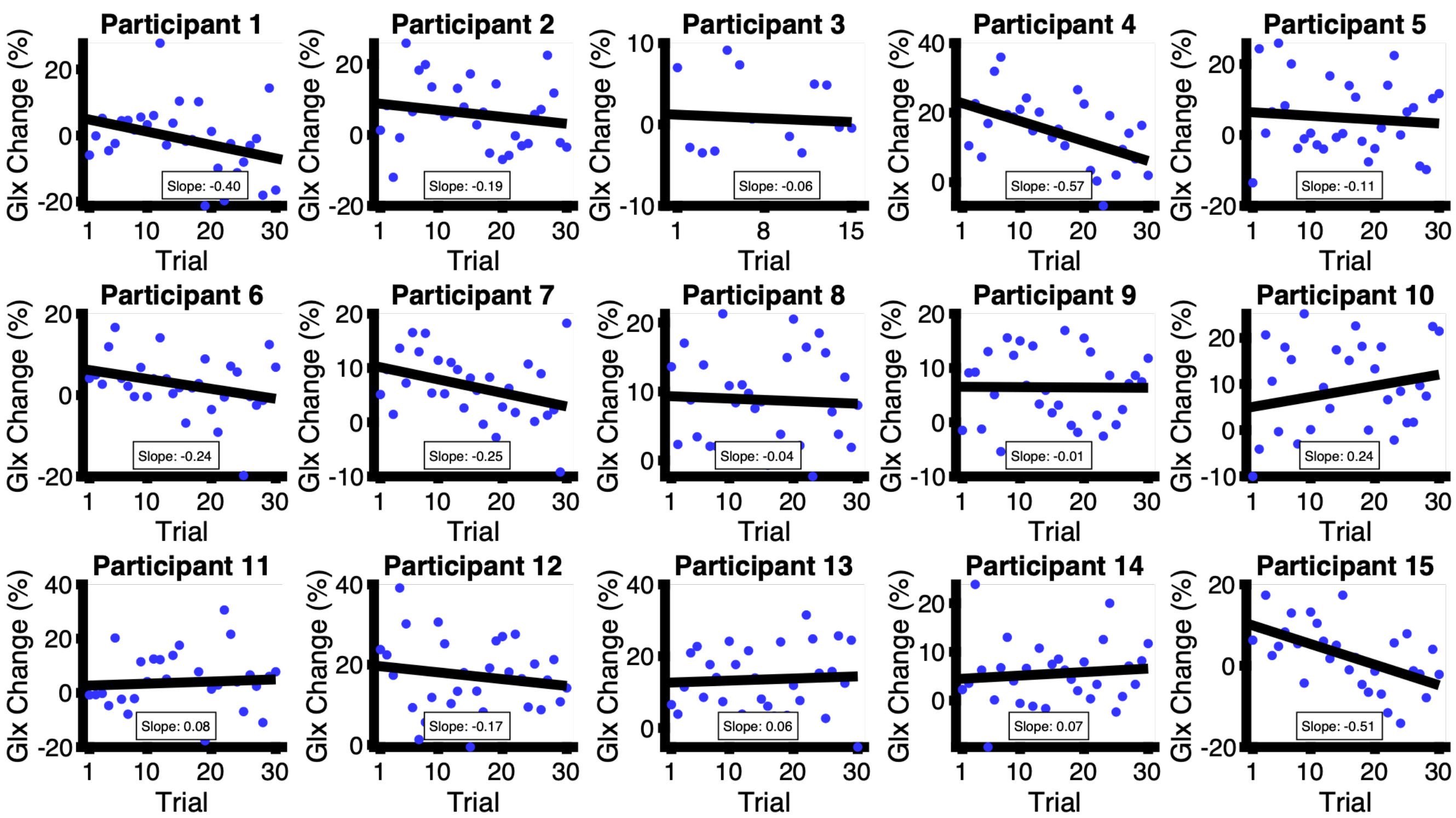

Participant 1
Slope: -0.40
Participant 2
Slope: -0.19
Participant 3
Slope: -0.06
Participant 4
Slope: -0.57
Participant 5
Slope: -0.11
Participant 6
Slope: -0.24
Participant 7
Slope: -0.25
Participant 8
Slope: -0.04
Participant 9
Slope: -0.01
Participant 10
Slope: 0.24
Participant 11
Slope: 0.08
Participant 12
Slope: -0.17
Participant 13
Slope: 0.06
Participant 14
Slope: 0.07
Participant 15
Slope: -0.51
Glx Change (%)
Trial
