# Supplementary material for: Real‐Time Neurofeedback Training to Modulate Glutamate‐Glutamine Concentration Using Functional Magnetic Resonance Spectroscopy
Source: NMR Biomed. 2026 Sep 21;39(11):e70399. doi: 10.1002/nbm.70399 (PMC13593363; doi:10.1002/nbm.70399)
Supplement: Supplementary file 6 [file NBM-39-e70399-s006.pdf]

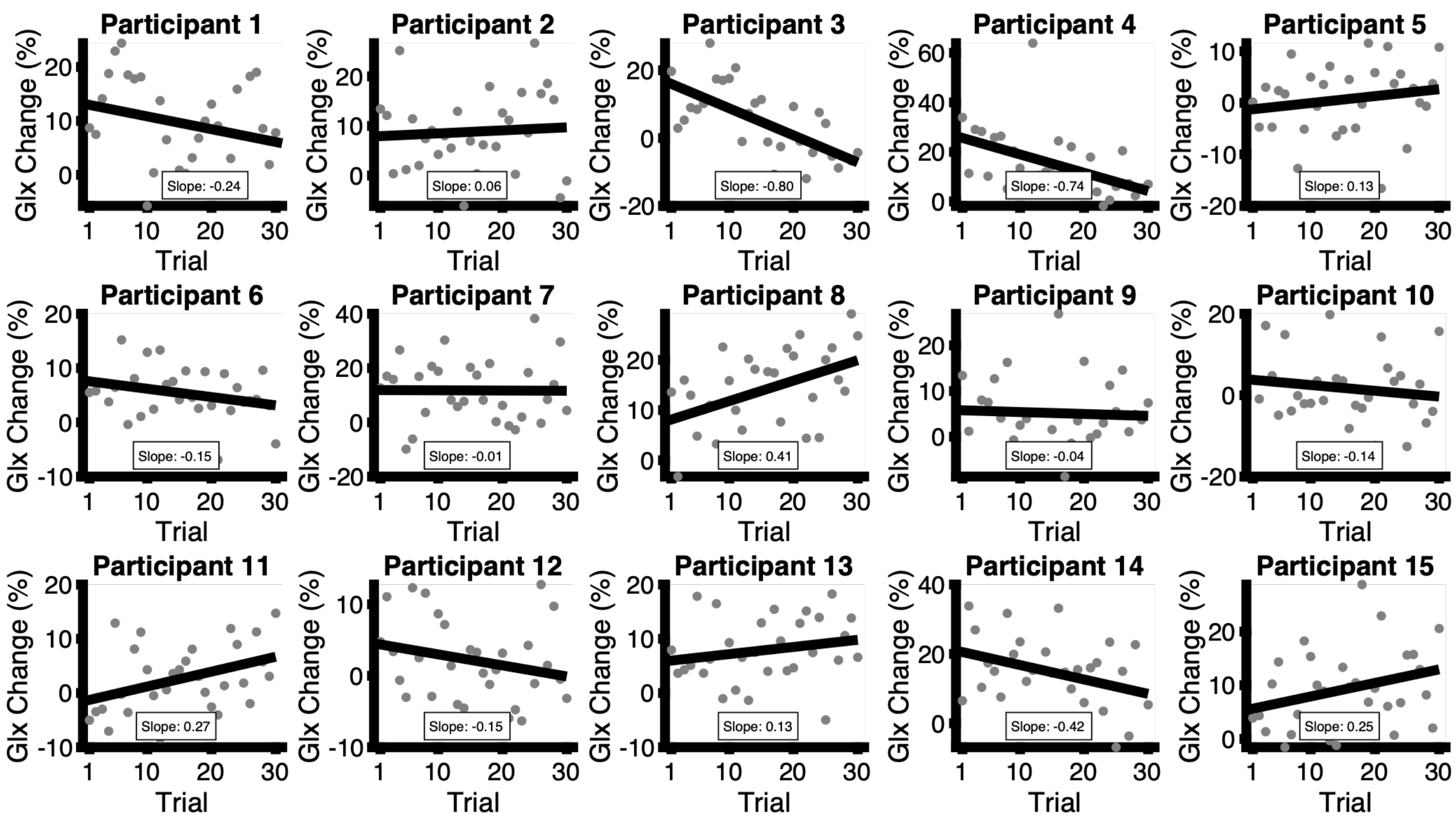

Participant 1
Glx Change (%)
Slope: -0.24
Trial
Participant 2
Slope: 0.06
Participant 3
Slope: -0.80
Participant 4
Slope: -0.74
Participant 5
Slope: 0.13
Participant 6
Slope: -0.15
Participant 7
Slope: -0.01
Participant 8
Slope: 0.41
Participant 9
Slope: -0.04
Participant 10
Slope: -0.14
Participant 11
Slope: 0.27
Participant 12
Slope: -0.15
Participant 13
Slope: 0.13
Participant 14
Slope: -0.42
Participant 15
Slope: 0.25
