# Supplementary material for: Real‐Time Neurofeedback Training to Modulate Glutamate‐Glutamine Concentration Using Functional Magnetic Resonance Spectroscopy
Source: NMR Biomed. 2026 Sep 21;39(11):e70399. doi: 10.1002/nbm.70399 (PMC13593363; doi:10.1002/nbm.70399)
Supplement: Supplementary file 8 [file NBM-39-e70399-s008.docx]

**Supplementary Material**

**Supplementary Table 1**. fMRS checklist proposed by Choi et al. (2021).

| **Site** (Name or Number): Brain Imaging Center at University of Regensburg / Bezirksklinikum Regensburg  1. Hardware |  |
| --- | --- |
| a. Field strength [T] | 3 T |
| b. Manufacturer | Siemens |
| c. Model (software version if available) | Prisma (Syngo MR XA30) |
| d. RF coils: nuclei (transmit/receive), number of channels, type, body part | 64 channel head/neck coil |
| e. Additional hardware | N/A |
| 2. Acquisition |  |
| a. Pulse sequence | Siemens PRESS sequence |
| b. Volume of Interest (VOI) locations | Early visual areas (V1 and V2) in occipital lobes |
| c. Nominal VOI size [cm^3^, mm^3^] | 2 x 2 x 2 cm |
| d. Repetition Time (TR), Echo Time (TE) [ms, s] | PRESS: TR 2000 ms; TE = 30 ms |
| e. Total number of Excitations or acquisitions per spectrum | For each of two consecutive fMRS runs: 16 acquisitions for water reference scan; 24 acquisitions for Glx reference scan; fifteen consecutive neurofeedback training trials with the following acquisitions per trial: 8 acquisitions for induction phase, 8 acquisitions for analysis phase, 8 acquisitions for feedback and rest phases |
| f. Additional sequence parameters (spectral width in Hz, number of spectral points, frequency offsets) | Spectral bandwidth: 1200 Hz; Spectral points: 1024 |
| g. Water Suppression Method | WET |
| h. Shimming Method, reference peak, and thresholds for “acceptance of shim” chosen | Automated B_0_ field mapping to < 20 Hz |
| i. Triggering or motion correction method | N/A |
| 3. Data analysis methods and outputs |  |
| a. Analysis software | LC Model Version 6.3-R; Osprey Version 2.4.0 |
| b. Processing steps deviating from quoted reference or product | Post-hoc analysis after data acquisition: Lorentzian linebroadening to the spectra to  account for linewidth changes due to the blood-oxygenation level dependent effect prior to LC model processing |
| c. Output measure | Glx concentration normalized to NAA+NAAG concentration from the same spectrum; Glx concentration normalized to Cr+PCr concentration from the same spectrum |
| d. Quantification references and assumptions, fitting model assumptions | Basis set was imported from LC model software package and included the following spectra: Aspartate (Asp), Creatine (Cr), Phosphocreatine (PCr), γ-Aminobutyric Acid (GABA), Glucose (Glc), Glutamine (Gln), Glutamate (Glu), Glycerophosphocholine (GPC), Glutathione (GSH), myo-Inositol (Ins), L-Lactate (Lac), N-Acetylaspartate (NAA), N-Acetylaspartylglutamate (NAAG), scyllo-Inositol (Scyllo), Taurine (Tau) |
| 4. Data Quality |  |
| a. Reported variables | CRLB, FWHM, S/N |
| b. Data exclusion criteria | Online: Shimming >= 20 Hz; Post-hoc analysis after data acquisition: for each participant, trial-wise induced percent Glx concentration changes from the reference concentration within a session using the three scaled median absolute deviation (MAD) away from the median as outlier criterion |
| c. Quality measures of postprocessing Model fitting | CRLB, FWHM, S/N |
| d. Sample Spectrum | See Supplementary Figures 1-3 |

**Supplementary Table 2.** Participants’ strategies during induction. Strategy category numbers correspond to the following: 1 = Emotions, inner experience; 2 = Social and autobiographical life; 3 = Sports, games, physical action; 4 = Sensory, nature, places; 5 = Objects, activities, knowledge.

| **Experiment 1 Experimental Group** | | |
| --- | --- | --- |
| *Participant* | *Strategy Description: Thinking about …* | *Strategy Categories* |
| *1* | shopping, people, especially faces, animals, flowers rising, glass of water filling, smells | 2, 4, 5 |
| *2* | historical events, coins | 5 |
| *3* | events of the last 24 hours, people and places from personal surroundings, scenes in these places | 2, 4 |
| *4* | actions, scenes from football, video games | 3, 4, 5 |
| *5* | playing tennis | 3 |
| *6* | solving mathematical problems, childhood home | 2, 5 |
| *7* | solving mathematical problems, singing a song | 5 |
| *8* | solving mathematical problems, personal memories | 1, 2, 5 |
| *9* | work, solving mathematical problems | 2, 5 |
| *10* | personal memories, daily routine | 1, 2 |
| *11* | memories of vacation, emotions | 1, 2 |
| *12* | action sports | 3, 4 |
| *13* | visual autobiographical memories of the recent past | 1, 2, 4 |
| *14* | world map with countries and their capitals, football games in the stadium, geopolitics | 3, 4, 5 |
| *15* | playing video games, video clips, daily routine, pets, cooking | 2, 3, 4 ,5 |
| **Experiment 1 Control Group** | | |
| *Participant* | *Strategy Description: Thinking about …* | *Strategy Categories* |
| *1* | emotions, hobbys, family, friends, travel, smell of forest, cooking, sea, music | 1, 2, 4, 5 |
| *2* | blowing up balloon that gets bigger or smaller (like green circle), something flying higher and higher, inflating a hot air balloon, becoming faster | 3, 4 |
| *3* | travel, work, visit friends, positive memories, details | 1, 2 |
| *4* | daily routine | 2 |
| *5* | positive and negative emotions, breathing and concentrating, content in different languages | 1, 5 |
| *6* | countries of the world and capitals, athletic exercises | 3, 4, 5 |
| *7* | skiing, playing football, mathematical problems, studying, playing chess, a whirlpool with a massage, going for a walk in the forest | 2, 3, 4, 5 |
| *8* | vacation, sport, surfing, holidays, summer, gymnastics | 2, 3, 4 |
| *9* | vacation, cooking, story telling from the third perspective, friends | 2, 4, 5 |
| *10* | future plans | 2 |
| *11* | doing things in order, conversation in English, purpose of the experiment | 2, 5 |
| *12* | family, animals | 2 |
| *13* | autobiographical memories, doing sports, family, friends | 1, 2, 3 |
| *14* | daily routine, future plans | 2 |
| *15* | emotions, physical sensations, consecutive letters of the alphabet, repetitive movements like stretching | 1, 3, 5 |
| **Experiment 2** | | |
| *Participant* | *Strategy Description: Thinking about …* | *Strategy Categories* |
| *1* | emotions, music videos | 1, 5 |
| *2* | sounds, sea, playing tennis | 3, 4 |
| *3* | numbers in different languages | 5 |
| *4* | action scenes, doing sports, playing chess, playing video games | 3, 4, 5 |
| *5* | doing sports, sounds, emotions, math calculations | 1, 3, 4, 5 |
| *6* | landscapes, summits, panorama, apartment tour, fast movements | 3, 4 |
| *7* | emotions, birthday party, marathon running | 1, 2, 3 |
| *8* | pets, imagination in multiple senses, touching fur | 4 |
| *9* | memories with family and friends | 1, 2 |
| *10* | animals, baking cookies, playing computer games, emotions | 1, 3, 5 |
| *11* | objects, tour the zoo, cooking, shopping, driving | 4, 5 |
| *12* | pets, faces, objects, inner monologue with yourself, alphabet, mindwandering | 1, 5 |
| *13* | thinking about recent events, distant past, social situations | 1, 2 |
| *14* | vacation by the sea, social situations, music, pets, plants, animals, sports, family, work | 1, 2, 3, 4, 5 |
| *15* | songs | 5 |

**Supplementary Figure 1.** PRESS spectra for induction trials in the experimental group in the first experiment. Spectra were corrected using Lorentzian linebroadening prior to LC model fitting. Each induction trial is indicated by the position on the y-axis in ascending order, with the bottom line corresponding to the first induction trial. Each spectrum shows the mean across partcipants. The shaded area represents the standard-error-of-the-mean (SEM). Glx between 2.1 and 2.5 ppm and at 3.75 ppm. N-acetyl-aspartate at 2 ppm.

**Supplementary Figure 2.** Spectra for induction trials in the control group in the first experiment. Otherwise same as Supplementary Figure 1.

**Supplementary Figure 3.** Spectra for induction trials in the second experiment. Otherwise same as Supplementary Figure 1.

**Supplementary Figure 4.** Difference spectra (induction minus reference) before (top row) and after correction (bottom row) using Lorentzian linebroadening. For each fMRS run and participant, the reference scan spectrum was subtracted from the mean spectrum across induction trials. The resulting difference spectra were first averaged across fMRS runs within each participant and subsequently across participants. The shaded area shows SEM. “Increase”: experimental group in the first experiment. “Control”: control group in the first experiment. “Decrease”: second experiment.

**Supplementary Figure 5.** Trial-by-trial results in the experimental group in the first experiment without Lorentzian linebroadening and without metabolite concentration outlier removal. Otherwise same as Figure 4.

**Supplementary Figure 6.** Trial-by-trial results in the control group in the first experiment. Otherwise same as Supplementary Figure 5.

**Supplementary Figure 7.** Trial-by-trial results in the second experiment. Otherwise same as Supplementary Figure 5.
